# Using valence and arousal scoring in speech to detect depressive symptoms: A study on family caregivers

**DOI:** 10.1371/journal.pone.0359556

**Published:** 2026-10-14

**Authors:** Mike K. P. So, Jenny T. Y. Tsang, Lupe S. H. Chan, Jacky N. L. Chan, Agnes Tiwari, Amanda M. Y. Chu

**Affiliations:** 1 Department of Information Systems, Business Statistics and Operations Management, The Hong Kong University of Science and Technology, Hong Kong; 2 School of Nursing, Tung Wah College, Hong Kong; 3 Department of Social Sciences and Policy Studies, The Education University of Hong Kong, Hong Kong; 4 Department of Information Systems, Business Statistics and Operations Management, The Hong Kong University of Science and Technology, Hong Kong; 5 School of Nursing, Hong Kong Sanatorium & Hospital, Hong Kong; 6 Department of Social Sciences and Policy Studies, The Education University of Hong Kong, Hong Kong; Catholic University of the Sacred Heart: Universita Cattolica del Sacro Cuore, ITALY

## Abstract

**Background:**

Depression is a prevalent mental health condition that significantly affects individuals’ well-being and daily functioning, making early depressive symptom detection crucial.

**Objectives:**

This study explores the potential of advancing speech analytics with dimensional sentiment analysis, using valence and arousal scoring to enhance depressive symptom detection in family caregivers.

**Methods:**

We recruited 116 family caregivers and assessed their depression levels using the Beck Depression Inventory-II. Participants provided verbal responses to 12 open-ended questions related to family resilience. The extracted words from the responses were analyzed for sentiment using the Chinese Valence-Arousal Words dictionary to determine their valence and arousal ratings. For each participant, we computed various summary statistics (e.g., mean, variance, quantiles) and frequency-domain statistics (e.g., spectral entropy, dominant frequency components) of valence and arousal distributions. These features were then used as predictors in nine different machine learning models evaluated under repeated 5-fold nested cross-validation with cost-sensitive cutoff selection, enabling robust classification of depression levels while prioritizing the identification of true positive cases.

**Results:**

Our analysis showed that participants with higher depressive symptoms used words with more extreme valence and arousal scores than those with lower depressive symptoms. The models demonstrated strong predictive utility of these emotional dimensions: the quadratic discriminant analysis (QDA) model achieved an out-of-sample sensitivity of 0.725, a specificity of 0.563, and an AUC of 0.7. These findings support that valence and arousal analysis provides meaningful discriminative power for identifying depressive symptoms. Importantly, the high sensitivity achieved by these models ensures that individuals with elevated symptoms are more reliably detected, thereby reducing the risk of missed cases. Overall, the results suggest strong potential for automated depressive symptom detection based on emotional signal analysis, with cost-sensitive optimization reducing false negatives while maintaining clinically relevant specificity.

**Conclusions:**

This study offers proof-of-concept evidence that emotional sentiment analysis of speech using valence and arousal scoring can capture linguistic markers associated with depressive symptoms among family caregivers. With further refinement and validation, this approach lays an important foundation for the development of more robust machine learning models and ultimately contributes to non-intrusive mental health screening tools.

## Introduction

Depression, also known as depressive disorder, is one of the most prevalent mental health conditions and a significant contributor to global disability [1]. It is characterized by a persistent low mood and a loss of interest in previously enjoyed activities. Additionally, it disrupts thinking, concentration, memory, and sleep patterns, all of which adversely impact daily functioning [2]. In severe cases, depression can lead to suicidal thoughts and behaviors [3]. According to the World Health Organization, approximately 5% of the global adult population is affected by depression, equating to around 280 million people [4]. Furthermore, over 700,000 individuals die by suicide each year, highlighting the critical nature of this condition [5]. In 2019, depression accounted for 584 disability-adjusted life years (DALYs) per 100,000 people worldwide, indicating its substantial contribution to the global burden of disease [6].

Identifying depressive symptoms and support for individuals with depression can significantly reduce the likelihood of developing severe depressive conditions and related illnesses [7]. However, more than 50% of potential depression cases remain undetected in high-income countries, with even lower detection rates in low- and middle-income countries [8]. A major obstacle to early depressive symptom identification is the limited accessibility of mental health service providers. Additionally, the cumbersome and subjective processes used for assessment hinder effective identification. Currently, the gold standard for depression assessment involves lengthy interviews conducted by specialists to thoroughly evaluate individuals’ psychosocial status using validated questionnaires [9]. These assessments heavily depend on the subjective responses of clients, which can affect the accuracy of the evaluations. Stigma surrounding mental health issues may cause individuals to provide socially acceptable answers instead of honest ones [10,11]. Furthermore, many individuals may not possess complete self-awareness, making it difficult for them to report their feelings accurately [12]. As a result, recognizing subtle signs of depression often relies on the evaluator’s expertise, which can influence both the efficiency and precision of the assessments. Therefore, there is an immediate need for alternative approaches that can complement existing methods and potentially circumvent some of these limitations.

In recent years, machine learning (ML) has emerged as a powerful tool for the automatic detection of mental health conditions [13]. Various ML models have been developed to identify mental health conditions through the analysis of a variety of features, including serum proteomics data [14,15], magnetic resonance imaging [16], electroencephalography [17], and electrodermal activity [18]. However, these approaches are often invasive or obtrusive in data collection, hindering the adaptability of mental health screening for larger populations. ML models utilizing data collected through non-invasive methods, such as social media activity, have also been proposed [19]. However, social media users tend to be more proficient with technology, making them unrepresentative of the general population. This can introduce noise and irrelevance in the analysis, limiting the accuracy of depressive symptom identification.

Speech analytics has gained attention in regard to depression assessment due to its convenient and non-invasive data collection methods, as well as the strong connection between speech and psychosocial status. The process of speech production involves a complex interplay of musculoskeletal and neurological systems that manage auditory, somatosensory, and visual information, along with language comprehension and production [20]. This complexity makes speech an information-rich signal, conveying unique thoughts and emotions that can serve as behavioral markers for assessing depression in the general population [21]. With recent developments in natural language processing (NLP) techniques, individuals’ speech can be automatically transcribed into text for linguistic analysis. This data can be utilized for the development of ML models that can automatically detect mental health conditions [22].

Existing ML models focusing on linguistic analysis typically examine word frequency and topic modeling [23]. Word frequency counts the occurrence of specific terms [24], with research indicating that terms such as “sad” and “hopeless” appear more frequently in the speech of individuals with depression [25]. However, relying solely on word occurrence without considering the meanings and emotional connotations of words can lead to suboptimal assessments of depression. Topic modeling identifies latent themes based on the co-occurrence of patterns of words [26]. For example, the theme “emotional distress” may emerge from clusters of terms such as “hopeless,” “worthless,” “empty,” “miserable,” and “crying.” While topic modeling provides a richer sentiment analysis of speech, it often requires manual labeling by researchers, which can be subjective and biased [27]. Additionally, themes may only capture categorized sentiment status, such as negative or positive emotions, limiting the nuance of sentiment identification. Consequently, suboptimal speech analytics restrict the accuracy of existing ML models.

Accurate depressive symptom detection relies on effective sentiment identification from speech, highlighting the need for a novel ML approach. We aim to explore the potential of developing a new ML methodology for depressive symptom detection that optimizes sentiment detection by incorporating insights from recent research on dimensional sentiment analysis. This kind of research evaluates emotional connotations of words across dimensions such as valence, arousal, dominance, and threat, using continuous numerical values [28,29]. Among these dimensions, valence and arousal are the most extensively studied [30]. This predominance is largely attributable to the influential circumplex model of affect from psychology, which posits that these two dimensions can sufficiently characterize a wide range of emotional states [31]. Valence reflects pleasantness or unpleasantness, while arousal indicates excitement or calmness. Both valence and arousal are evaluated using a nine-point scale, where a score of 1 indicates the most negative state, 9 indicates the most positive state, and 5 is designated as neutral. The valence-arousal (VA) rating of words can be represented as points on the VA coordinate plane presented in Fig 1 [32]. The horizontal axis represents the valence rating, while the vertical axis represents the arousal rating. Each word is rated by multiple annotators in the Self-Assessment Manikin (SAM) scale [33], yielding the mean and standard deviation of the valence and arousal ratings. The mean score represents the overall level of valence or arousal of a word, while a larger standard deviation indicates a greater level of disagreement among the raters. This VA approach has led to the creation of sentiment dictionaries, such as the National Research Council Canada’s Valence, Arousal, and Dominance (NRC-VAD) Lexicon, which includes VA ratings for 20,000 English words [34], and the Chinese Valence-Arousal Words (CVAW) dictionary, with 5,512 Chinese words [32].

**Fig 1:**
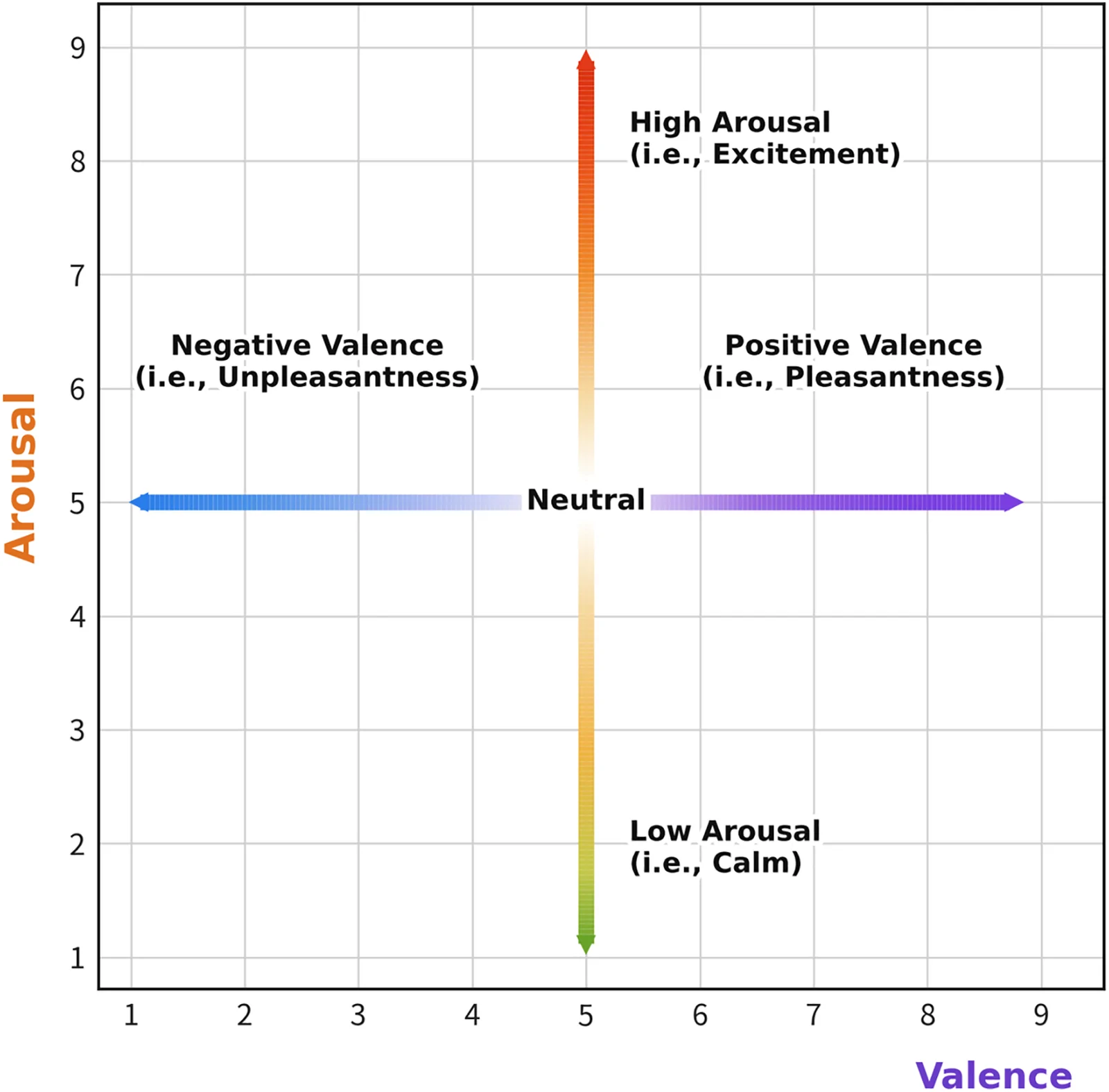
The VA coordinate plane that represents the valence-arousal (VA) rating of words.

In this paper, we develop machine learning models that use summary statistics of the valence and arousal scores derived from participants’ speech as predictors. We apply our machine learning models to a group of 116 family caregivers recruited at a non-profit organization providing integrated family and community services in Hong Kong. Family caregivers are particularly vulnerable to depression due to the considerable stress associated with the physical, emotional, and financial demands of caregiving [35]. Recognizing the signs of depression and implementing supportive measures can help preserve caregivers’ psychosocial health and overall quality of life. Furthermore, in the data collection process, we utilize a concise set of targeted questions that are related to family resilience rather than direct symptom queries, potentially reducing stigma and social desirability bias. Our findings provide preliminary evidence that this approach may differentiate between varying levels of depressive symptoms that caregivers experience, while also emphasizing the importance of considering the sentiment context of words for effective speech analysis. With further refinement and validation in larger, more diverse populations, this approach holds potential to inform the development of more robust machine learning models for non-intrusive mental health screening.

## Methods

### Participants

Family caregivers with diverse backgrounds were recruited from a community center operated by a non-profit organization providing integrated family and community services in Hong Kong. Participants were included if they: (1) were aged 18 years or older; (2) self-identified as family caregivers providing at least 14 hours per week of unpaid care to one or more family members (e.g., older adults, spouses, children, or other relatives); (3) were able to provide informed consent; and (4) were capable of communicating verbally in Cantonese. Participants were excluded if they had a history of severe psychiatric disorders (e.g., schizophrenia) or cognitive impairments (e.g., dementia) that would prevent them from completing the interview or the study instruments, as determined by the registered social workers at the community center [36,37]. A total of 116 family caregivers were approached and recruited between 1 August 2018 and 31 July 2019 and the demographic characteristics of the participants are presented in Table 1**.** Registered social workers explained the study to all potential participants and obtained signed informed consent.

**Table 1:** Demographic characteristics of the participants.

Characteristic	Category	*n* (%)
**Gender**	Female	114 (98.3)
	Male	2 (1.7)
**Age group**	20–30 years	17 (14.7)
	31–40 years	56 (48.3)
	41–50 years	20 (17.2)
	51–60 years	14 (12.1)
	61 years or above	9 (7.8)
**Marital status**	Married or cohabiting	96 (82.8)
	Single	5 (4.3)
	Separated, widowed, or divorced	15 (12.9)
**Employment status**	Not employed	94 (81.0)
	Employed	22 (19.0)
**Primary care recipient(s)**	Child/Children	102 (87.9)
	Spouse	32 (27.6)
	Elderly family member(s)	17 (14.7)
	Other family member(s)	16 (13.8)
	**Total**	**116 (100)**

All participants were assessed for depressive symptoms using the Beck Depression Inventory II (BDI-II), a validated 21-item self-report instrument designed to measure the severity of depression in adolescents and adults [38]. In this study, we employed a validated Chinese version of the BDI-II, which demonstrated good internal consistency (Cronbach’s alpha = 0.85 [39]). Participants rated each item on a four-point Likert scale ranging from 0 (“absence of the symptom”) to 3 (“full manifestation of the symptom”). Total scores range from 0 to 63, with higher scores reflecting more severe depressive symptoms.

Following established guidelines [40,41], we applied a cutoff score of 20 on the BDI-II to categorize participants: those scoring below 20 were assigned to the low depressive symptom group, and those scoring 20 or above were assigned to the high depressive symptom group. While registered social workers assisted with recruitment and eligibility screening, the definitive group assignment was based solely on the BDI-II cutoff. Accordingly, 84 participants were categorized into the low depressive symptom group, and 32 participants were categorized into the high depressive symptom group.

Notably, the current study represents a secondary exploratory analysis of data originally collected to develop a conversation-based depression screening tool for non-clinical staff in community settings. While the primary objective focused broadly on speech analytics (including linguistic and acoustic features), this analysis specifically focuses on emotional signals.

### Ethics statement

This project was approved by the Human Participants Research Panel of The Hong Kong University of Science and Technology (reference no. 252). All participants received detailed information about the study’s aims and procedures and provided informed consent. Participants had the right to withdraw from the study at any time, and participation was entirely voluntary.

### Study procedure

All participants were asked 12 open-ended questions about their family circumstances. Their responses were recorded for subsequent analysis and for the development of machine learning models. Open-ended questions were chosen for this study because prior research indicates that they yield richer emotional insights compared to structured speech tasks [42]. These questions were crafted to explore dimensions of family resilience, as previous studies have shown a correlation between family resilience and depression, with higher resilience linked to lower levels of depression [43,44]. The 12 questions are detailed in Table A in S1 Text and encompass three essential aspects of Walsh’s family resilience theory: family belief systems, organizational patterns, and communication patterns [45]. Family belief systems represent the ability to cope with crises by finding meaning in adversity, maintaining a positive outlook, and fostering spiritual beliefs. Organizational patterns reflect supportive family dynamics that are flexible, interconnected, and capable of accessing social networks and economic resources. Communication patterns emphasize the effectiveness of family members in engaging in clear dialogue, promoting emotional expression, and collaborating in regard to problem-solving during challenging times. These questions focus on family resilience and daily life rather than directly querying depressive symptoms, which may potentially reduce stigma and social desirability bias while providing insight into participants’ levels of depressive symptoms, given the established association between family resilience and depression [43,44]. All 116 participants answered the 12 questions, and their verbal responses were recorded for subsequent analysis.

### Statistical analysis

The workflow of speech analytics is depicted in Fig 2. The analysis consists of three major components: speech processing and word extraction, word sentiment, and an integrative analysis that combines word sentiment and word count for the classification of depressive symptom levels utilizing machine learning models.

**Fig 2:**
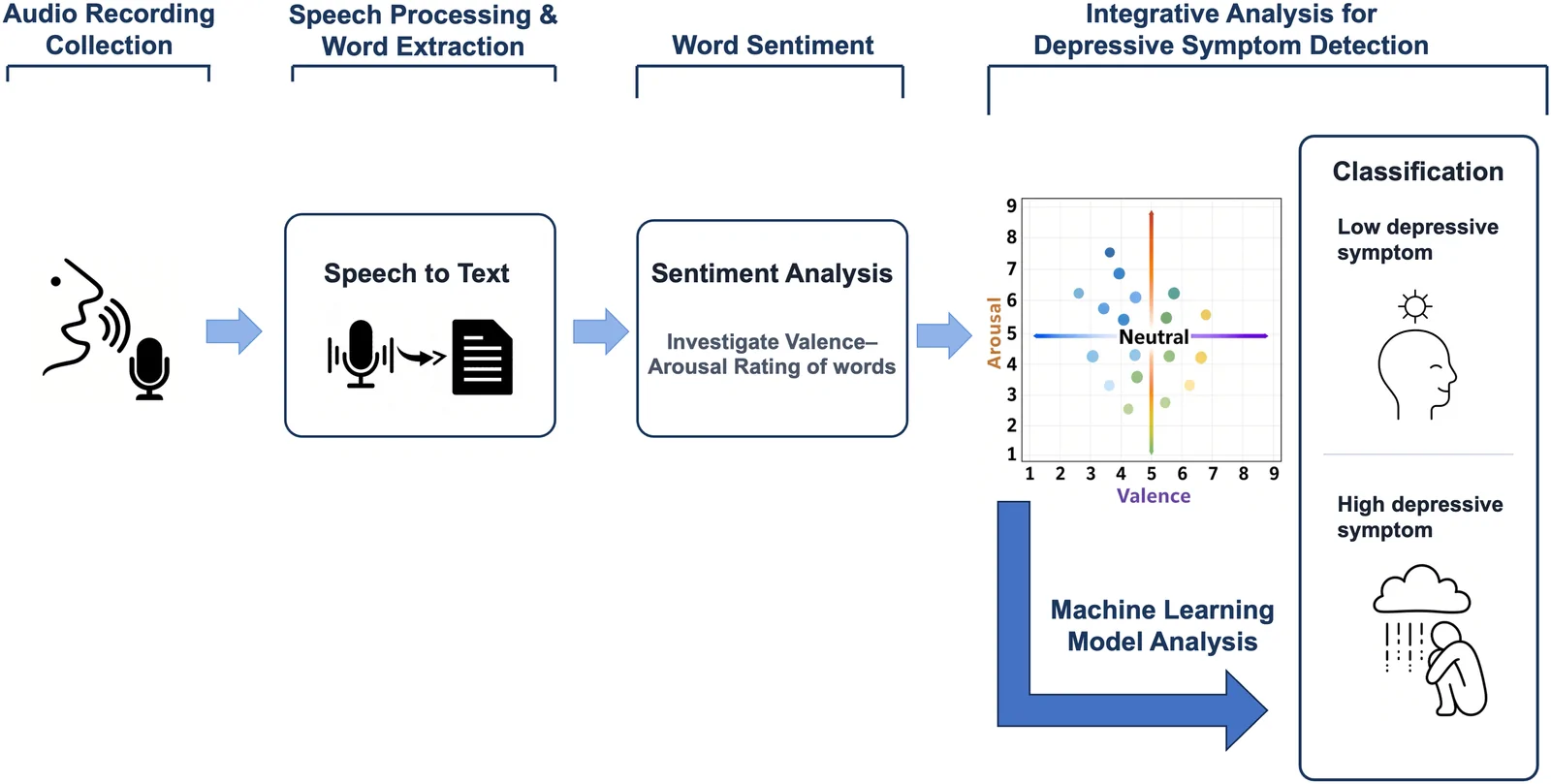
Workflow diagram of the proposed speech analytics process.

#### Speech processing and word extraction.

Following the collection of audio recordings from family caregivers, we converted their speech into text transcripts using automatic speech recognition (ASR). Interview recordings were first converted to a standardized single-channel 16 kHz format using the *librosa* library. Transcription was then performed with the Qwen3-ASR speech-to-text model (Qwen/Qwen3-ASR-1.7B), with language detection set to automatic mode to accommodate both Cantonese and English speech within a unified workflow. The resulting transcripts were normalized from Simplified to Traditional Chinese characters using the *OpenCC* library (s2t configuration) to ensure stylistic and linguistic consistency. Normalized text was subsequently segmented into discrete sentences via regular expressions based on sentence-final punctuation, establishing clear boundaries for downstream processing. For word-level analysis, the segmented text was further tokenized into individual word tokens using the *PyCantonese* library [46]. Full implementation details of the pipeline are provided in S2 Text, while the manual validation of ASR transcription and written translation are described in S3 and S4 Texts.

#### Word sentiment, negation, and gradable adverb analysis.

While mean valence and arousal ratings provide a useful baseline, they cannot fully capture the complexity of emotional expression in natural language. Negation (e.g., “not happy,” “never satisfied”) can invert or diminish sentiment, while gradable adverbs (e.g., “very,” “slightly,” “extremely”) scale its intensity. Relying solely on dictionary averages risks misrepresenting the intended emotional tone and overlooking subtle gradations in how caregivers articulate distress.

To address these limitations, we propose a methodology that integrates both variability and modifier effects into the sentiment framework. Using regression analysis, mean valence, and arousal ratings will be adjusted by predictors for negation and gradable adverbs, producing context-sensitive scores that better reflect the emotional meaning conveyed in speech.

The Chinese Valence–Arousal Profile (CVAP) [32] already extends coverage by including some modified forms, such as “有點不開心” (slightly unhappy) and “超不舒服” (very uncomfortable). However, its scope remains incomplete; for example, expressions like “沒有不開心” (not unhappy) are absent. To enhance generalizability, we propose a negation model in which the effects of negation are estimated from CVAP data and systematically applied across CVAW entries, thereby broadening coverage of negated forms.

Similarly, gradable adverbs such as “有點” (slightly), “非常” (very), and “極度” (extremely) are critical for capturing emotional intensity. For instance, “非常開心” (very happy) conveys stronger positivity than “開心” (happy), while “有點不舒服” (slightly uncomfortable) softens negativity.

By integrating both negation and gradable adverbs into regression-based adjustments, we refine dictionary-based sentiment scores into context-sensitive measures, enabling a more accurate representation of emotional expression.

##### Negation and gradable adverb models.

Each token was matched against CVAW to retrieve baseline valence and arousal ratings. Negation markers were then detected and applied to subsequent words. For valence, we adopted the following transformation:


$$\begin{array}{c} v^{\prime }=c_{v}+\gamma \cdot (v-c_{v})+\gamma_{\text{soft}}I_{\text{soft}}(v-c_{v})+\gamma_{\text{str}\text{ength}}I_{\text{str}\text{ength}}(v-c_{v})+\gamma_{\text{soft},\text{0}}I_{\text{soft}}+\gamma_{\text{str}\text{ength},\text{0}}I_{\text{str}\text{ength}}, \end{array}$$

where v is the CVAW valence score of the base word and $v^{\prime }$ is the adjusted valence score of its negated form in the CVAP. $I_{\text{soft}}=\text{1}$ if the modifier is softening (e.g., “not very,” “slightly not”), $I_{\text{str}\text{ength}}=\text{1}$ if the modifier is strengthening (e.g., “very not,” “extremely not”), and 0 otherwise.

For arousal, the transformation is:


$$\begin{array}{c} a^{\prime }=a+\beta \cdot (a-c_{a})+\beta_{\text{soft}}I_{\text{soft}}(a-c_{a})+\beta_{\text{str}\text{ength}}I_{\text{str}\text{ength}}(a-c_{a})+\beta_{\text{soft},\text{0}}I_{\text{soft}}+\beta_{\text{str}\text{ength},\text{0}}I_{\text{str}\text{ength}}, \end{array}$$

where a is the CVAW arousal score of the base word and $a^{\prime }$ is the adjusted arousal score of its negated form in the CVAP.

A naïve approach would set γ=β=1, assuming negation always produces a perfect symmetric flip around the neutral midpoint (for valence) and a full shift toward neutrality (for arousal). However, empirical studies show that negation effects are not uniform. Psycholinguistic evidence indicates that negation often dampens rather than fully reverses emotional polarity, and that arousal shifts vary depending on context and marker type [47].

##### Gradable adverb model (without negation).

Gradable adverbs also play a crucial role in shaping emotional intensity, even when no explicit negation is present. Words such as “very,” “slightly,” or “extremely” amplify or hedge emotional expressions, altering both valence and arousal dynamics. For example, “very happy” conveys stronger positivity than “happy,” while “slightly uncomfortable” softens negativity. Incorporating these modifiers ensures that the model captures both intensification and attenuation effects, providing a more nuanced representation of emotional variation in natural language.

The valence gradable adverb model is given by:


$$\begin{array}{c} v^{\prime }=c_{v}+\gamma \cdot (v-c_{v})+\gamma_{\text{soft}}I_{\text{soft}}(v-c_{v})+{\gamma_{\text{str}\text{ength}}I}_{\text{str}\text{ength}}(v-c_{v})+\gamma_{\text{soft},\text{0}}I_{\text{soft}}+\gamma_{\text{str}\text{ength},\text{0}}I_{\text{str}\text{ength}}, \end{array}$$

where v is the CVAW valence score of the base word and $v^{\prime }$ is the adjusted valence score after applying a gradable adverb.

The arousal gradable adverb model is:


$$\begin{array}{c} a^{\prime }=a+\beta \cdot (a-c_{a})+{\beta_{\text{soft}}I}_{\text{soft}}(a-c_{a})+\beta_{\text{str}\text{ength}}I_{\text{str}\text{ength}}(a-c_{a})+\beta_{\text{soft},\text{0}}I_{\text{soft}}+\beta_{\text{str}\text{ength},\text{0}}I_{\text{str}\text{ength}}. \end{array}$$

#### Valence–arousal feature construction.

Beyond simple averages of valence and arousal scores, we extended our analysis to a richer set of statistical descriptors designed to capture both distributional and frequency‑domain characteristics of caregiver speech. For each valence and arousal series, we computed the standard deviation and percentile measures (5th, 25th, 75th, and 95th percentiles), which highlight extremes in linguistic behavior and may signal unusually long sentences or complex dependency structures indicative of heightened cognitive load or emotional strain.

To further characterize temporal dynamics, we constructed time series of valence and arousal scores based on the sequential order of speech. From these series, we derived spectral features that capture rhythmic and structural properties of language, including dominant frequency, spectral centroid, spectral entropy, low‑to‑high power ratio, total spectral power, and numbers of high arousal spikes and negative valence spikes. We also calculated approximate entropy to quantify the regularity, unpredictability, and complexity of fluctuations, and examined the synchrony correlation between valence and arousal trajectories to assess how emotional dimensions co‑vary over time.

A variable selection procedure was then applied to identify predictors that differed significantly between low and high symptom groups. To mitigate multicollinearity, predictors with high correlations were removed when they exhibited smaller *z*‑values relative to competing variables.

Taken together, these features provide complementary insights: quantile measures emphasize extremes and variability in linguistic behavior, while spectral and entropy measures capture the underlying rhythm, concentration, and unpredictability of sentence construction [48]. This multidimensional representation moves beyond mean values, enabling our models to more effectively characterize the complexity of caregiver speech and its association with depression severity. By integrating distributional, temporal, and synchrony‑based features, the framework offers a more nuanced lens on emotional expression, strengthening its utility for early depressive symptom detection.

## Results

### Exploratory analysis

A total of n=116 family caregivers participated in this study. All participants completed the BDI-II [38] to help us understand their level of depressive symptoms. Among the 116 participants, 84 scored below the BDI-II screening threshold of 20 and were categorized as the low depressive symptom group (low BDI-II group). The remaining 32 participants scored at or above the threshold and were categorized as the high depressive symptom group (high BDI-II group). No statistically significant differences were observed between the two groups regarding demographic data, including age, education, and gender. In addition to completing the BDI-II, all participants verbally answered 12 family resilience-related questions in interviews guided by registered social workers, and their responses were recorded for subsequent analysis.

After speech processing and word extraction, we investigated word sentiment. For sentiment analysis, the extracted words are compared to those in the CVAW dictionary [32,49]. A total of 977 words identified in participants’ speech are found in the CVAW dictionary. The median number of these words per participant is 54, with counts varying from a minimum of 28 to a maximum of 264. The valence and arousal ratings of these words, as reported in the CVAW dictionary, are used for subsequent analysis.

The 20 most and least frequently appearing words in each of the high and low BDI-II groups are listed in Table 2, along with their valence ratings. Only unique words are included; specifically, these words appear in either the high depressive symptom group or the low depressive symptom group, but not in both. Notably, the unique words with the most negative valence ratings in the high depressive symptom group include many terms associated with hopelessness, such as “disheartened,” “unfortunate,” “inferiority,” and “not as good as,” and words reflecting the complexity of life and death, including “stroke,” “suicide,” “feeling unwell,” “pass away,” and “decline.” Participants in the high depressive symptom group also tend to use words with extreme valence ratings, such as “great luck,” which has a valence rating of 8.9, and “stroke,” which has a valence rating of 1.4. In contrast, participants in the low BDI-II group generally refrain from using highly extreme words and exhibit a lower frequency of mentioning terms related to hopelessness and death.

**Table 2:** The 20 most extreme negative and positive valence words in each of the high and low BDI-II groups, along with their corresponding valence ratings. Only unique words are included; specifically, these words appear in either the high depressive symptom group or the low depressive symptom group, but not in both.

Unique Words in High depressive symptom Group		Unique Words in Low depressive symptom Group	
Most positive words	Most negative words	Most positive words	Most negative words
大吉 Great luck (8.9)	中風 Stroke (1.4)	大好 Excellent (8.5)	砍頭 Behead (1.6)
完美 Perfect (7.6)	變態 Pervert (1.7)	稱職 Competent (8)	受害 Victimized (1.8)
精彩 Brilliant (7.6)	離譜 Outrageous (1.9)	信任 Trust (7.8)	怨恨 Resentment (2)
寶貝 Treasure (7.4)	自殺 Suicide (2)	極好 Extremely good (7.8)	心碎 Heartbroken (2.2)
爽快 Refreshed (7.4)	難受 Feeling unwell (2)	豐盛 Sumptuous (7.7)	絕望 Despair (2.2)
戰勝 Defeat (7.4)	不孝 Unfilial (2.1)	負責任 Responsible (7.7)	火大 Furious (2.2)
興奮 Excited (7.4)	虐待 Abuse (2.2)	閃耀 Shining (7.6)	嚇人 Scary (2.3)
大喜 Great joy (7.4)	不及 Not as good as (2.2)	感謝 Grateful (7.4)	吵死 Noisy (2.3)
厲害 Awesome (7.3)	衰落 Decline (2.3)	贏得 To win (7.4)	仇 Hatred (2.4)
知己 Confidant (7.2)	心死 Disheartened (2.3)	勇敢 Brave (7.4)	失控 To lose control (2.4)
愛人 Lover (7.2)	過世 Pass away (2.4)	美滿 Prosperous (7.4)	放肆 Unbridled (2.4)
幸運 Lucky (7.2)	嫌棄 To disdain (2.4)	智慧 Wisdom (7.4)	怒 Anger (2.4)
偉大 Great (7.2)	兇惡 Vicious (2.4)	歡喜 Joyful (7.4)	怒氣 Wrath (2.4)
陽光 Sunshine (7.2)	出錯 To make a mistake (2.5)	長壽 Longevity (7.4)	憂鬱 Depressed (2.4)
歡樂 Joy (7.2)	不幸 Unfortunate (2.6)	叫好 To applaud (7.4)	淒涼 Desolate (2.4)
盡力而為 To do one’s best (7.2)	無理 Unreasonable (2.6)	自給自足 Self-sufficient (7.4)	餓死 To starve to death (2.4)
伴侶 Partner (7)	討厭 To hate (2.6)	舒暢 Agreeably (7.3)	悲哀 Sad (2.6)
受惠 To benefit (7)	自卑 Inferiority (2.6)	紓解 To relieve (7.3)	難熬 Hard to endure (2.6)
高興 Delighted (7)	批判 To criticize (2.6)	贏過 To beat (7.3)	抱怨 To complain (2.8)
愛護 To cherish (7)	惡劣 Vile (2.6)	乾淨 Clean (7.3)	放縱 To indulge (2.8)

Fig 3 illustrates the distributions of the valence and arousal scores, respectively, grouped by depressive symptom level. In Fig 3(a), we observe that the speech of participants in the high depressive symptom group (colored in orange) has, on average, lower valence scores compared to those in the low depressive symptom group (colored in blue). A similar pattern is also observed for the distributions of arousal ratings presented in Fig 3(b), in which the speech of participants in the high depressive symptom group has, on average, lower arousal scores than that of those in the low depressive symptom group, although this difference was less pronounced than that observed in the valence scores. To visualize the distributions clearly, Fig 4 contains the boxplots for both valence and arousal scores, grouped by depressive symptom level. We observe that the distributions of valence scores for the high and low BDI-II groups are different.

**Fig 3:**
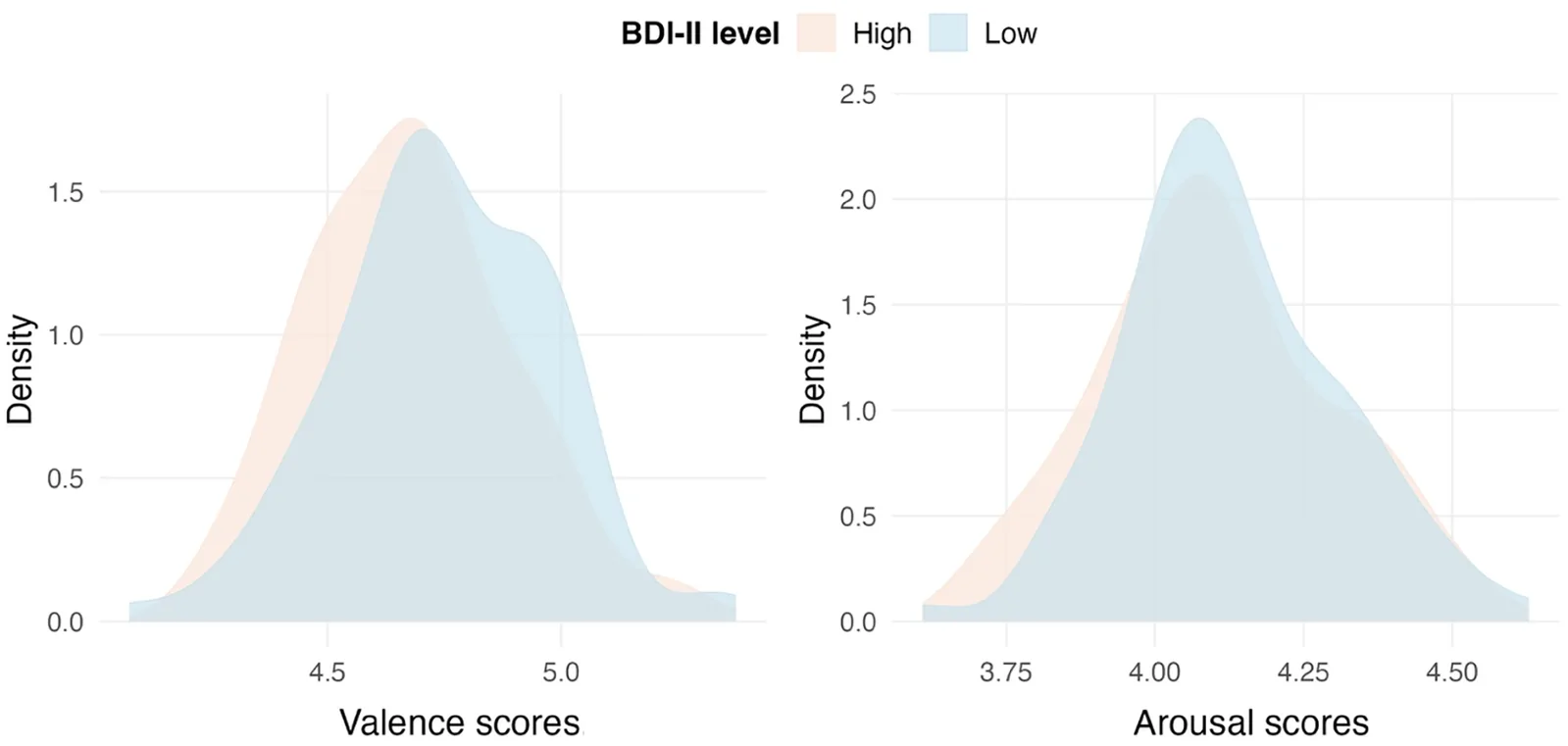
The distributions of the (a) valence and (b) arousal scores, grouped by BDI-II level. The low depressive symptom group is colored in blue, and the high depressive symptom group is colored in orange. ***(a) Valence scores. (b) Arousal scores.***

**Fig 4:**
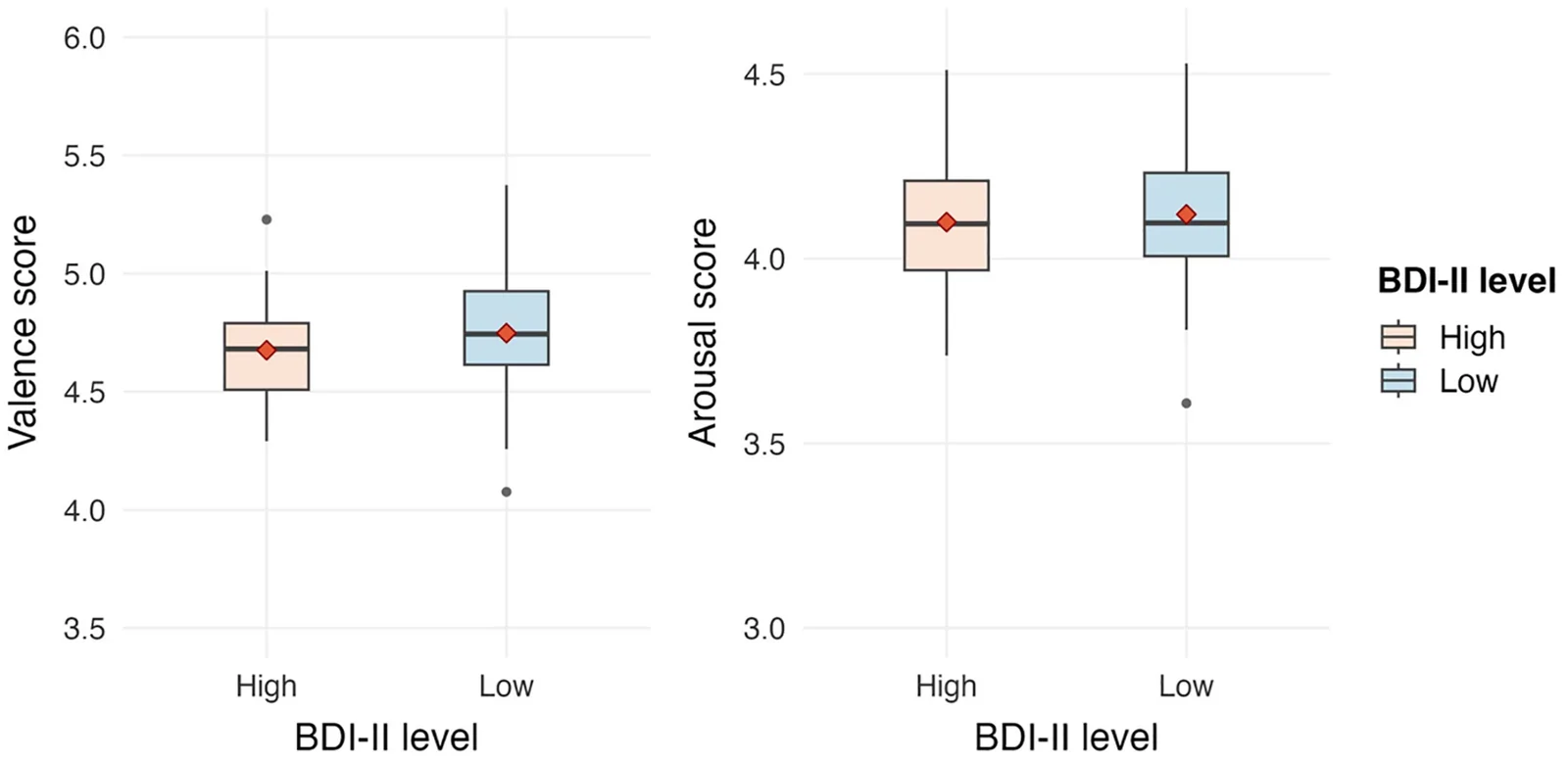
The boxplots illustrate the distributions of the mean valence and arousal scores for the low depressive symptom group (represented in blue) and the high depressive symptom group (represented in orange). The red dot within each boxplot represents the average value of the arousal or valence scores for the respective groups.

The two-sample *t*-test for the difference in mean valence scores between the high and low BDI-II groups was not statistically significant, t-value = −1.55, p = 0.124 (95% CI [–0.165, 0.020]), with a small effect size (Cohen’s *d* = –0.32, 95% CI [–0.73, 0.09]). In contrast, the difference in mean arousal scores between the high and low depressive symptom groups was even less pronounced and clearly non-significant, t-value = −0.71, p = 0.482 (95% CI [–0.103, 0.049]), whose Cohen’s *d* = –0.15, (95% CI [–0.55, 0.26]).

In the basic analysis using mean valence and mean arousal scores, the group differences were not statistically significant. Traditional mean-based features may not fully capture the subtle emotional fluctuations associated with depression. To illustrate this, we examined two additional dynamic features that better reflect emotional instability and reactivity:

- **Approximate Entropy of valence (ApEn)**: This quantifies the unpredictability and complexity of the valence time series. Higher ApEn indicates more irregular, less predictable emotional fluctuations (emotional instability), whereas lower ApEn reflects more rigid or monotonic emotional patterns.
- **High-arousal spikes**: This counts the number of times where arousal exceeds the 95th percentile of an individual’s own arousal distribution. It captures moments of intense emotional reactivity or sudden high-arousal bursts, which may be more frequent in individuals with higher depressive symptoms even when average arousal levels are similar.

Fig 5 contains the boxplots for both Approximate Entropy of valence and High-arousal spikes, grouped by BDI-II level. We observe that the distributions of the high and low depressive symptom groups are clearly different: the high depressive symptom group shows higher ApEn of valence and a greater number of High-arousal spikes.

**Fig 5:**
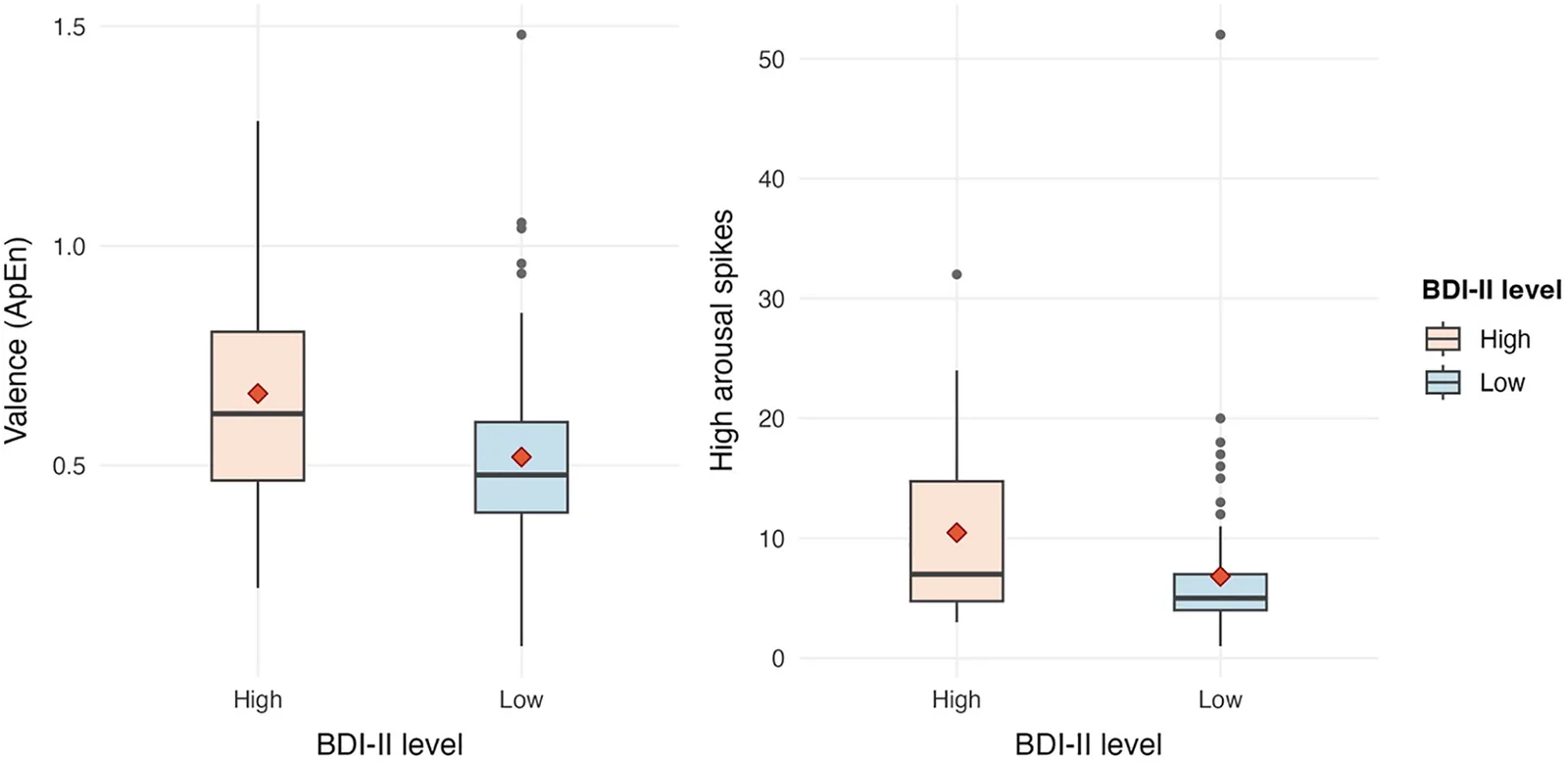
The boxplots illustrate the distributions of the Approximate Entropy (ApEn) of valence and High-arousal spikes for the low depressive symptom group (represented in blue) and the high depressive symptom group (represented in orange). The red dot within each boxplot represents the average value of ApEn of valence and High-arousal spikes for the respective groups.

The difference in Approximate Entropy of valence between the high and low BDI-II groups was statistically significant at the 0.05 level, *t*-value = 2.92, p = 0.004 (95% CI [0.047, 0.243]), demonstrating a medium effect size (Cohen’s *d* = 0.61, 95% CI [0.19, 1.02]). Similarly, the difference in the number of high-arousal spikes between the high and low depressive symptom groups was also statistically significant, *t*-value = 2.66, p = 0.009 (95% CI [0.929, 6.365]), with a medium effect size (Cohen’s *d* = 0.55, 95% CI [0.14, 0.96]).

To investigate these findings further, Fig 6 illustrates the joint distribution of the mean valence and arousal scores of the participants, colored by BDI-II level. The figure also includes the 99% confidence contours of the fitted bivariate normal distributions for both low depressive and high depressive symptom groups, with the high depressive symptom group represented by the blue contour and the low depressive symptom group by the red contour. We observe that the two joint distributions are located in two distinct positions. Furthermore, the mixed pattern of the scatter points for the high and low BDI-II groups suggests that these two groups cannot be effectively separated using a linear classification approach. Therefore, we also consider non-linear machine learning models to capture the non-linearity within the classification model. The findings indicate an association between lower VA ratings and higher depression levels, highlighting the potential of utilizing word sentiment features to detect depressive symptoms.

**Fig 6:**
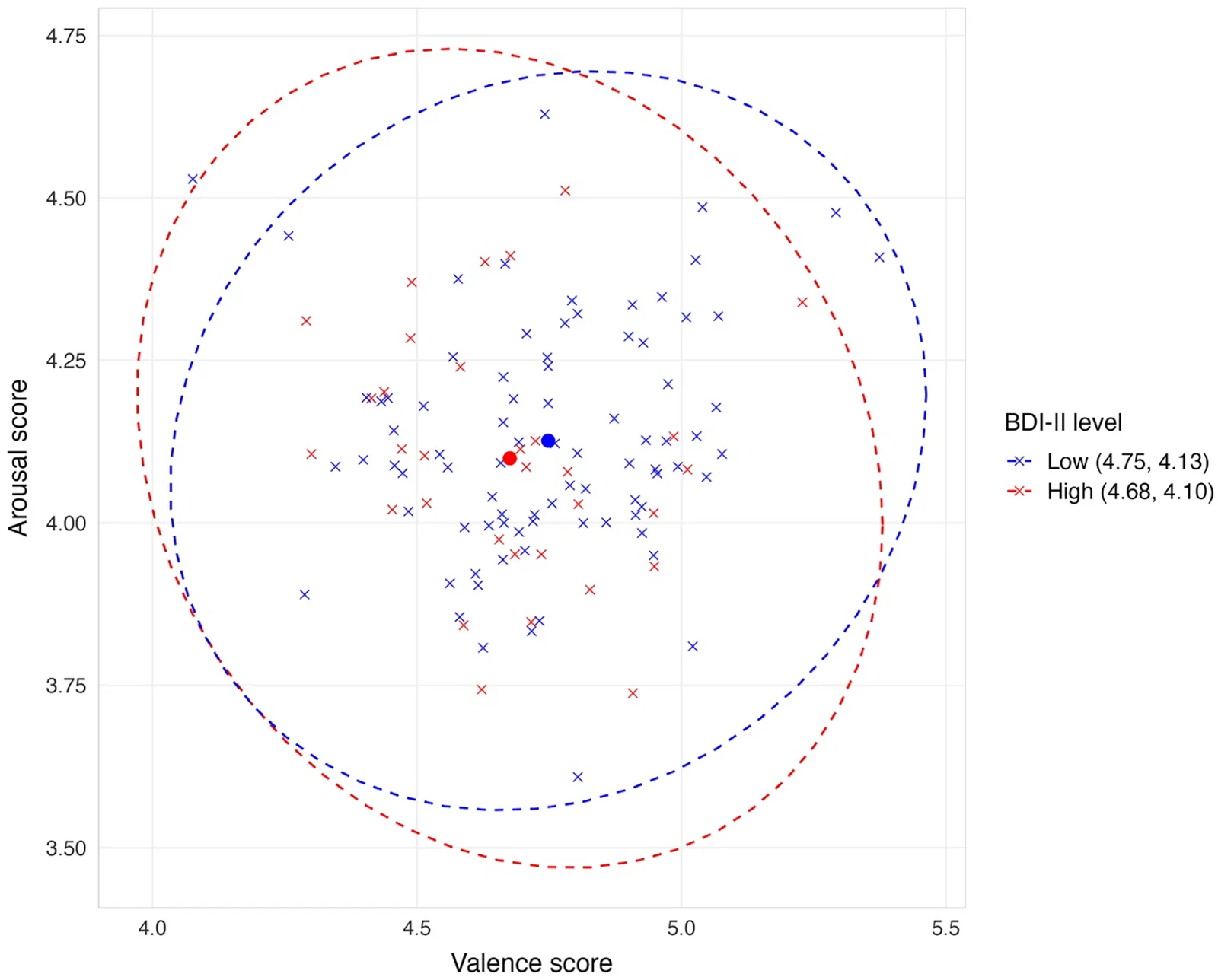
A two-dimensional plot illustrating the joint distribution of the valence and arousal scores for the low and high depressive symptom groups. Each point in the plot represents a participant, with low depressive symptom participants indicated by blue crosses and high depressive symptom participants indicated by red crosses. The mean valence and arousal score for the low depressive symptom group is depicted as a solid blue dot, while a solid red dot represents the mean score for the high depressive symptom group. The dashed circles depict the 99% confidence contours for the fitted bivariate normal distributions of the low depressive symptom group (in blue) and the high depressive symptom group (in red).

We are also interested in comparing the speech behaviors of participants with low and high levels of depression. This analysis aims to provide insights into the usefulness of word sentiment in depressive symptom detection. Fig 6 presents two-dimensional plots that illustrate the valence and arousal ratings of the words used by two selected participants. Fig 7(a) depicts a participant with a high level of depressive symptom (a BDI-II score of 37), while Fig 7(b) represents a participant with a low level of depressive symptom (a BDI-II score of 16).

**Fig 7:**
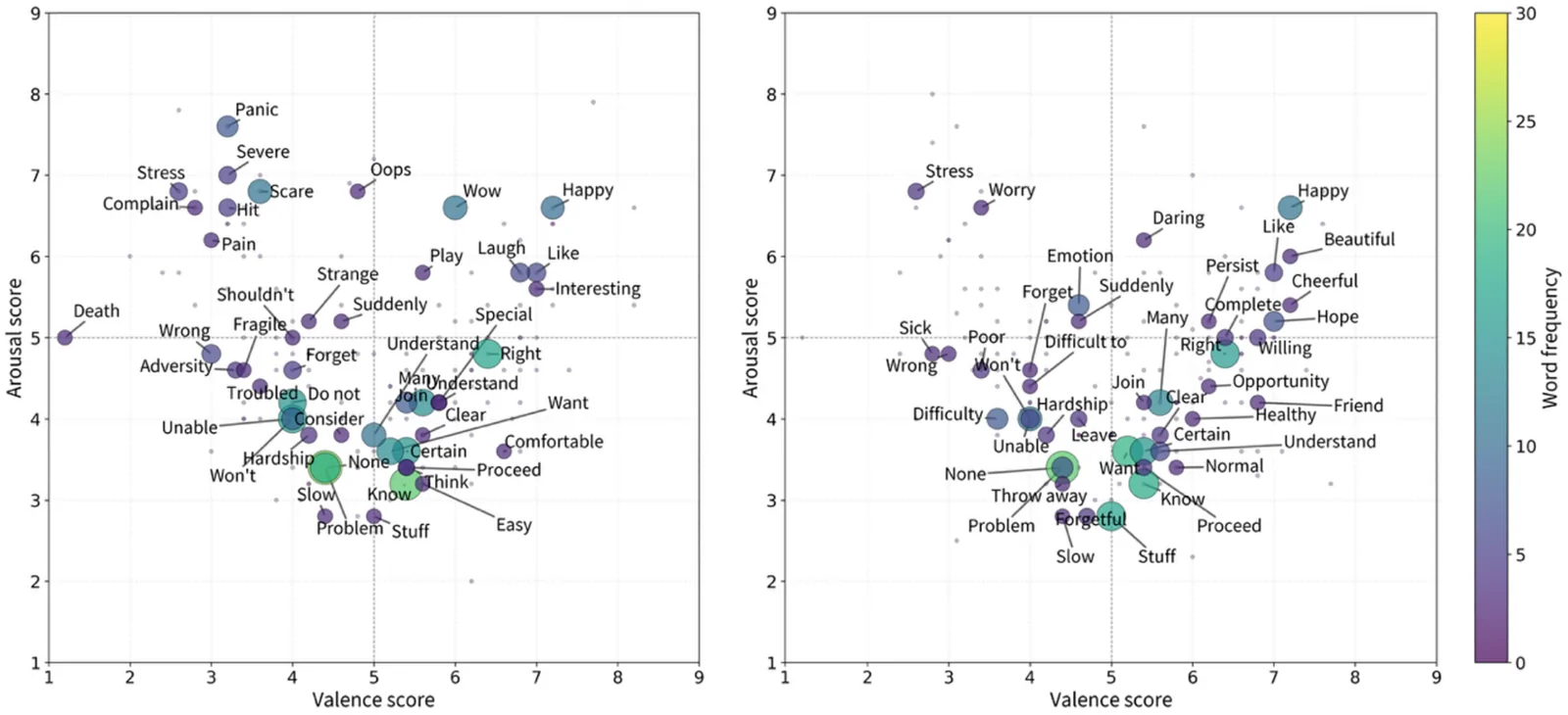
The two-dimensional plots depict the valence and arousal ratings for words used by two selected participants: (a) a participant exhibiting a high level of depressive symptoms (BDI-II score of 37) and (b) a participant exhibiting a low level of depressive symptoms (BDI-II score of 16). The frequency of each word in their speech is represented by a color scale shown in the figures. Only words with frequencies of three or more are included in the plots, while words with a frequency of two are indicated by grey points. (a) The participant with high depressive symptoms. (b) The participant with low depressive symptoms.

The frequency of each word in the participants’ speech is represented using the color scale indicated in the figures. Words that appear more than once in the transcripts, corresponding to the displayed points, are included in the figures. Fig 7(a) illustrates that the participant with a high level of depressive symptom tends to use words with extreme negative arousal and valence ratings, such as “death,” “pain,” “pressure/stress,” and “complain.” In contrast, Fig 7(b) reveals that the participant with a low level of depressive symptom tends to mention words with non-extreme arousal and valence ratings. These figures highlight a significant distinction in speech behaviors between the two participants. The findings suggest that there are notable variations in speech patterns between individuals with low and high levels of depressive symptoms, indicating the potential to utilize word sentiment as indicators in the identification of depressive symptoms.

### Negation and adverb model results

#### Negation models.

Table 3 reports the fitted parameters for the valence and arousal negation models. The main negation effect (γ=−0.425,p<0.001) indicates that negation dampens emotional polarity rather than fully reversing it. Softening modifiers (e.g., “not very,” “slightly not”) produced a significant reduction in valence ($\gamma_{\text{soft}}=\text{0}.\text{3}\text{1}\text{0}$, p<0.001), while strengthening modifiers showed no reliable slope effect ($\gamma_{\text{strength}}=-\text{0}.\text{0}\text{4}\text{6}$, p>0.05). However, intensified negation led to a marked drop in valence ($\gamma_{\text{strength},\text{0}}=-\text{0}.\text{4}\text{7}\text{4}$, p<0.001).

**Table 3:** Fitted parameters for the negation models.

	Estimate	Std. Error	t value	Pr(>|t|)	
(a) Valence negation model.					
$c_{v}$	4.600	0.041	111.853	< 0.001	***
γ	−0.425	0.031	−13.529	< 0.001	***
$\gamma_{\text{soft}}$	0.310	0.037	8.468	< 0.001	***
$\gamma_{\text{strength}}$	−0.046	0.038	−1.215	0.225	
$\gamma_{\text{soft},\text{0}}$	−0.073	0.058	−1.247	0.213	
$\gamma_{\text{strength},\text{0}}$	−0.474	0.074	−6.406	< 0.001	***
(b) Arousal negation model.					
$c_{a}$	4.964	0.083	59.553	<0.001	***
β	−0.720	0.056	−12.756	<0.001	***
$\beta_{\text{soft}}$	0.107	0.064	1.654	0.0984	.
$\beta_{\text{strength}}$	0.024	0.065	0.365	0.7155	
$\beta_{\text{soft},\text{0}}$	−0.376	0.060	−6.248	<0.001	***
$\beta_{\text{strength},\text{0}}$	0.890	0.067	13.239	<0.001	***

For arousal, negation shifted arousal toward the center (β=−0.720, p<0.001), but effects varied by modifier type. Softening negation produced a significant intercept reduction ($\beta_{\text{soft},\text{0}}=-\text{0}.\text{3}\text{7}\text{6}$, p<0.001), while strengthening negation increased arousal substantially ($\beta_{\text{strength},\text{0}}=\text{0}.\text{8}\text{9}\text{0}$, p<0.001). These results suggest that negation generally neutralizes arousal, but intensified forms heighten emotional salience.

#### Gradable adverb models.

Table 4 presents the fitted parameters for the valence and arousal gradable adverb models. The main effect (γ=0.952, p<0.001) shows that valence is strongly preserved, with gradable adverbs scaling intensity rather than reversing polarity. Softening adverbs (e.g., “slightly,” “somewhat”) reduced valence strength ($\gamma_{\text{soft}}=-\text{0}.\text{1}\text{4}\text{1}$, p<0.001), while strengthening adverbs (e.g., “very,” “extremely”) amplified valence substantially ($\gamma_{\text{strength}}=\text{0}.\text{4}\text{2}\text{3}$, p<0.001).

**Table 4:** Fitted parameters for the gradable adverb models.

	Estimate	Std. Error	t value	Pr(>|t|)	
(a) Valence gradable adverb model.					
$c_{v}$	3.128	1.110	2.819	0.005	**
γ	0.952	0.021	44.818	<0.001	***
$\gamma_{\text{soft}}$	−0.141	0.028	−5.047	<0.001	***
$\gamma_{\text{strength}}$	0.423	0.027	15.619	<0.001	***
$\gamma_{\text{soft},\text{0}}$	0.203	0.195	1.041	0.298	
$\gamma_{\text{strength},\text{0}}$	−0.787	0.431	−1.825	0.068	.
(b) Arousal gradable adverb model.					
$c_{a}$	7.580	0.394	19.259	<0.001	***
β	−0.149	0.023	−6.406	<0.001	***
$\beta_{\text{soft}}$	−0.055	0.034	−1.625	0.104	
$\beta_{\text{strength}}$	−0.014	0.033	−0.423	0.672	
$\beta_{\text{soft},\text{0}}$	−0.511	0.093	−5.503	<0.001	***
$\beta_{\text{strength},\text{0}}$	0.717	0.082	8.783	<0.001	***

For arousal, the main effect was negative (β=−0.149, p<.001), indicating that gradable adverbs tend to pull the rating closer to the center. Slope effects for softening and strengthening were not significant, but intercept shifts were significant: softening adverbs lowered arousal ($\beta_{\text{soft},\text{0}}=-\text{0}.\text{5}\text{1}\text{1}$, p<0.001), while strengthening adverbs elevated it ($\beta_{\text{strength},\text{0}}=\text{0}.\text{7}\text{1}\text{7}$, p<0.001).

The R-squared values for the valence and arousal negation models were 0.52 and 0.51, respectively, indicating that these models explain just over half of the variance in emotional ratings. By contrast, the valence and arousal gradable adverb models achieved higher explanatory power, with R-squared values of 0.85 and 0.72. This marked improvement suggests that gradable adverbs provide a stronger and more consistent adjustment to valence and arousal estimation than negation, capturing a greater proportion of the variability in emotional expression.

### Machine learning model performance metrics

To evaluate the classification performance, we conduct repeated 5-fold cross-validation with 100 independent repetitions. In each repetition, the 5-fold cross-validation process randomly divides the dataset into five equally sized partitions or folds. The machine learning models are trained on four of these folds (the training set), and the classification performance is evaluated using the remaining fold (the test set). This process is repeated 5 times, such that each fold serves as the test set exactly once. We perform 5-fold cross-validation for 100 iterations to mitigate the effects of random partitioning on the dataset and to provide a more robust estimate of classification performance.

To evaluate the performance of our model on a test set, we utilize the following metrics: ROC-AUC (the area under the receiver operating characteristic curve) and accuracy [50]. Accuracy is defined as the proportion of correct predictions relative to the total number of cases examined. The formula for accuracy can be represented as


$$\begin{array}{c} \text{Accuracy}=\frac{\text{TP}+\text{TN}}{\text{TP}+\text{TN}+\text{FP}+\text{FN}}, \end{array}$$ (1)

where TP, FP, TN, and FN denote the counts of true positives, false positives, true negative, and false negatives, respectively.

Furthermore, to evaluate the performance of our model in predicting positives and negatives separately, we use the following metrics. The true positive rate (TPR) refers to the proportion of actual positive cases that are correctly identified by the model, and is given by


$$\begin{array}{c} \text{TPR}=\frac{\text{TP}}{\text{TP}+\text{FN}}. \end{array}$$ (2)

The true negative rate (TNR) measures the proportion of actual negative cases that are correctly identified. It is calculated as follows:


$$\begin{array}{c} \text{TNR}=\frac{\text{TN}}{\text{TN}+\text{FP}}. \end{array}$$ (3)

The positive predictive value (PPV) indicates the proportion of positive classification results that are true positives, and is defined as


$$\begin{array}{c} \text{PPV}=\frac{\text{TP}}{\text{TP}+\text{FP}}. \end{array}$$ (4)

The negative predictive value (NPV) indicates the proportion of negative classification results that are true negatives, and is defined as


$$\begin{array}{c} \text{NPV}=\frac{\text{TN}}{\text{TN}+\text{FN}}. \end{array}$$ (5)

These metrics presented in Equations (1) to (5) provide a comprehensive assessment of the classification performance of the proposed model.

It is important to note that several commonly reported performance metrics—including sensitivity, specificity, accuracy, PPV, and NPV—depend on the choice of classification cutoff. In this study, the cutoff was determined by minimizing the expected cost function on the validation set within the inner cross-validation loop. This procedure provides an adaptive balance between sensitivity and specificity while explicitly incorporating the asymmetric consequences of false negatives and false positives.

The expected cost function is defined as


$$\begin{array}{c} \text{Expected Cost}=C\cdot (\text{1}-\text{Sensitivity})\pi_{\text{1}}+(\text{1}-\text{Specificity})\pi_{\text{0}}, \end{array}$$

where $\pi_{\text{1}}$ and $\pi_{\text{0}}$ denote the prior probabilities of positive and negative cases, respectively. The parameter C reflects the relative penalty assigned to false negatives compared to false positives. In the context of early depressive symptom detection, it is natural to set C>1. A false negative corresponds to failing to identify an individual with elevated symptoms, which may delay intervention and increase the risk of adverse outcomes. By contrast, a false positive only triggers additional screening or evaluation, which carries comparatively minor consequences. The full derivation of the expected cost function is provided in S5 Text.

### Machine learning model performance results

To evaluate classification performance, we implemented nine widely used machine learning classifiers that represent both linear and non-linear approaches. These include logistic regression, linear discriminant analysis (LDA), and quadratic discriminant analysis (QDA) as representative linear models; support vector machines (SVM) and k-nearest neighbors (kNN) as margin-based and instance-based classifiers; and four ensemble or neural approaches—random forest, gradient boosting, extreme gradient boosting (XGB), and a multi-layer perceptron (MLP) classifier. This diverse set of models allows us to compare performance across different algorithmic families, ensuring that the observed results are not specific to a single modeling paradigm but reflect broader methodological robustness.

Table 5 summarizes the classification performance of multiple machine learning models under a cost-sensitive framework with C=4, reflecting the higher penalty assigned to false negatives. Across models, sensitivity values were consistently higher than specificity, confirming that the cost-weighted cutoff selection effectively prioritized the identification of positive cases. This is particularly important in the context of early depressive symptom detection, where minimizing missed cases is a clinical priority.

**Table 5:** Average performance metrics and 95% credible intervals (CIs) for nine machine learning classifiers under repeated 5-fold nested cross-validation with 100 independent repetitions. Models were evaluated using three predictor configurations: (1) valence-related statistics alone, (2) arousal-related statistics alone, and (3) combined valence and arousal statistics. Reported metrics include sensitivity, specificity, expected cost, AUC, accuracy, positive predictive value (PPV), negative predictive value (NPV), and F1-score. Within each repetition, the inner CV-folds were used to select the classification cutoff by minimizing the expected cost function, thereby adapting the threshold to balance sensitivity and specificity under asymmetric misclassification costs. A cost-sensitive penalty of C=4 was applied, reflecting the higher clinical importance of avoiding false negatives in early depressive symptom detection.

Model	Predictor	Sensitivity	Specificity	Expected cost	AUC	Accuracy	PPV	NPV	F1 score
Mode1 1: Logistic regression	Valence	0.785 (0.634, 0.942)	0.343 (0.143, 0.551)	1.522 (1.073, 2.012)	0.67 (0.605, 0.708)	0.464 (0.362, 0.594)	0.329 (0.263, 0.404)	0.528 (0.291, 0.836)	0.446 (0.375, 0.514)
Mode1 2: Random Forest		0.781 (0.653, 0.905)	0.396 (0.249, 0.56)	1.473 (1.053, 1.914)	0.639 (0.564, 0.717)	0.503 (0.42, 0.603)	0.338 (0.293, 0.4)	0.814 (0.601, 0.922)	0.462 (0.391, 0.522)
Mode1 3: MLP classifier		0.799 (0.61, 0.938)	0.383 (0.251, 0.526)	1.417 (0.93, 2.09)	0.648 (0.56, 0.73)	0.498 (0.415, 0.577)	0.336 (0.275, 0.397)	0.825 (0.584, 0.943)	0.465 (0.383, 0.536)
Mode1 4: XGB classifier		0.756 (0.618, 0.88)	0.413 (0.277, 0.527)	1.564 (1.067, 1.989)	0.632 (0.549, 0.698)	0.508 (0.43, 0.585)	0.336 (0.288, 0.392)	0.805 (0.637, 0.918)	0.454 (0.392, 0.514)
Mode1 5: Gradient boosting classifier		0.803 (0.656, 0.914)	0.309 (0.142, 0.453)	1.468 (1.047, 1.895)	0.604 (0.503, 0.684)	0.445 (0.346, 0.526)	0.311 (0.263, 0.359)	0.732 (0.461, 0.91)	0.44 (0.383, 0.491)
Mode1 6: Support vector machine		0.733 (0.46, 0.971)	0.324 (0.106, 0.569)	1.76 (1.029, 2.621)	0.584 (0.418, 0.691)	0.437 (0.328, 0.559)	0.299 (0.217, 0.386)	0.544 (0.261, 0.834)	0.404 (0.294, 0.471)
Mode1 7: kNN		0.775 (0.605, 0.914)	0.48 (0.332, 0.595)	1.42 (0.839, 2.044)	0.681 (0.624, 0.74)	0.561 (0.468, 0.629)	0.373 (0.311, 0.422)	0.869 (0.784, 0.944)	0.49 (0.409, 0.553)
Mode1 8: LDA		0.678 (0.586, 0.776)	0.555 (0.439, 0.668)	1.729 (1.407, 2.138)	0.682 (0.641, 0.711)	0.59 (0.51, 0.664)	0.379 (0.307, 0.459)	0.829 (0.776, 0.874)	0.472 (0.399, 0.528)
Mode1 9: QDA		0.763 (0.667, 0.876)	0.521 (0.417, 0.615)	1.416 (1.041, 1.748)	0.7 (0.648, 0.738)	0.588 (0.51, 0.638)	0.385 (0.324, 0.442)	0.866 (0.814, 0.921)	0.502 (0.438, 0.551)
Mode1 1: Logistic regression	Arousal	0.835 (0.634, 1)	0.195 (0, 0.379)	1.46 (0.982, 2.115)	0.637 (0.593, 0.665)	0.372 (0.276, 0.479)	0.285 (0.221, 0.337)	0.357 (0, 0.666)	0.411 (0.325, 0.452)
Mode1 2: Random Forest		0.76 (0.591, 0.936)	0.232 (0.041, 0.407)	1.738 (1.234, 2.33)	0.567 (0.498, 0.627)	0.377 (0.284, 0.463)	0.274 (0.23, 0.312)	0.436 (0.138, 0.749)	0.392 (0.327, 0.436)
Mode1 3: MLP classifier		0.733 (0.553, 0.931)	0.267 (0.05, 0.462)	1.817 (1.248, 2.373)	0.58 (0.494, 0.65)	0.396 (0.292, 0.501)	0.277 (0.224, 0.347)	0.52 (0.251, 0.796)	0.389 (0.328, 0.442)
Mode1 4: XGB classifier		0.724 (0.529, 0.871)	0.279 (0.099, 0.493)	1.852 (1.38, 2.436)	0.56 (0.489, 0.633)	0.402 (0.294, 0.525)	0.277 (0.223, 0.334)	0.546 (0.273, 0.793)	0.387 (0.313, 0.442)
Mode1 5: Gradient boosting classifier		0.82 (0.601, 1)	0.156 (0, 0.376)	1.564 (1, 2.147)	0.529 (0.454, 0.61)	0.339 (0.276, 0.438)	0.266 (0.217, 0.316)	0.284 (0, 0.589)	0.393 (0.316, 0.444)
Mode1 6: Support vector machine		0.852 (0.633, 1)	0.131 (0, 0.341)	1.467 (0.988, 2.135)	0.487 (0.361, 0.623)	0.329 (0.267, 0.446)	0.266 (0.205, 0.323)	0.219 (0, 0.476)	0.394 (0.309, 0.45)
Mode1 7: kNN		0.711 (0.572, 0.88)	0.34 (0.183, 0.534)	1.819 (1.335, 2.308)	0.601 (0.535, 0.65)	0.442 (0.355, 0.559)	0.306 (0.235, 0.377)	0.51 (0.272, 0.787)	0.404 (0.333, 0.478)
Mode1 8: LDA		0.734 (0.568, 0.876)	0.323 (0.176, 0.487)	1.731 (1.278, 2.211)	0.649 (0.623, 0.671)	0.436 (0.345, 0.526)	0.294 (0.238, 0.353)	0.691 (0.403, 0.882)	0.406 (0.346, 0.466)
Mode1 9: QDA		0.738 (0.578, 0.871)	0.306 (0.142, 0.479)	1.727 (1.291, 2.2)	0.642 (0.597, 0.676)	0.425 (0.336, 0.524)	0.29 (0.223, 0.343)	0.673 (0.406, 0.865)	0.402 (0.339, 0.461)
Mode1 1: Logistic regression	Valence and arousal	0.737 (0.56, 0.885)	0.468 (0.242, 0.668)	1.605 (1.084, 2.2)	0.669 (0.616, 0.71)	0.543 (0.38, 0.666)	0.367 (0.282, 0.457)	0.707 (0.437, 0.877)	0.472 (0.387, 0.547)
Mode1 2: Random Forest		0.761 (0.606, 0.881)	0.392 (0.229, 0.543)	1.559 (1.102, 2.049)	0.648 (0.583, 0.714)	0.494 (0.396, 0.595)	0.331 (0.284, 0.409)	0.791 (0.605, 0.903)	0.45 (0.386, 0.526)
Mode1 3: MLP classifier		0.747 (0.577, 0.902)	0.311 (0.157, 0.499)	1.708 (1.221, 2.253)	0.587 (0.504, 0.66)	0.431 (0.345, 0.543)	0.296 (0.253, 0.36)	0.678 (0.393, 0.879)	0.414 (0.353, 0.471)
Mode1 4: XGB classifier		0.727 (0.59, 0.871)	0.471 (0.263, 0.632)	1.617 (1.195, 2.191)	0.654 (0.583, 0.732)	0.542 (0.398, 0.647)	0.356 (0.269, 0.423)	0.807 (0.563, 0.897)	0.465 (0.381, 0.535)
Mode1 5: Gradient boosting classifier		0.782 (0.607, 0.931)	0.341 (0.146, 0.524)	1.506 (1.038, 2.083)	0.628 (0.559, 0.715)	0.462 (0.354, 0.567)	0.32 (0.266, 0.382)	0.681 (0.357, 0.89)	0.443 (0.383, 0.496)
Mode1 6: Support vector machine		0.765 (0.562, 0.964)	0.284 (0.071, 0.543)	1.666 (1.082, 2.38)	0.588 (0.424, 0.714)	0.417 (0.319, 0.549)	0.305 (0.241, 0.396)	0.44 (0.144, 0.808)	0.412 (0.327, 0.473)
Mode1 7: kNN		0.746 (0.591, 0.876)	0.509 (0.366, 0.632)	1.492 (1.084, 2.054)	0.692 (0.642, 0.736)	0.575 (0.494, 0.645)	0.378 (0.314, 0.441)	0.852 (0.738, 0.914)	0.488 (0.423, 0.551)
Mode1 8: LDA		0.689 (0.586, 0.78)	0.597 (0.479, 0.701)	1.646 (1.363, 2.054)	0.685 (0.652, 0.712)	0.623 (0.535, 0.69)	0.408 (0.331, 0.487)	0.837 (0.763, 0.882)	0.499 (0.419, 0.564)
Mode1 9: QDA		0.725 (0.597, 0.836)	0.563 (0.428, 0.663)	1.528 (1.129, 2)	0.696 (0.643, 0.737)	0.608 (0.517, 0.674)	0.4 (0.325, 0.471)	0.848 (0.688, 0.907)	0.503 (0.422, 0.565)

Among the single-predictor models, valence-based classifiers achieved high sensitivity but relatively low specificity (QDA achieved a sensitivity of 0.763 and a specificity of 0.521). Arousal-based models tended to yield even higher sensitivity but at the expense of specificity (LDA achieved a sensitivity of 0.734 while the specificity was 0.323). When valence and arousal predictors were combined, performance improved across several metrics. Sensitivity remained robust while specificity increased (QDA achieved a sensitivity of 0.725 and a specificity of 0.563; kNN achieved a sensitivity of 0.746 and a specificity of 0.509).

Notably, QDA using valence-related predictors alone incurred a lower expected cost (1.416) than QDA using both valence- and arousal-related predictors (1.528). This outcome reflects the higher sensitivity of the valence-based QDA model. Because the expected cost function applies a heavier penalty to false negatives, models with greater sensitivity are favored, even if their specificity is somewhat reduced.

Overall, Table 5 demonstrates that while cost-weighted cutoff selection increases sensitivity across all models, the combination of valence and arousal predictors provides a more stable balance between sensitivity and specificity. This supports the methodological choice to integrate both dimensions of emotional expression, and highlights the potential of discriminant-based approaches for achieving clinically meaningful performance in early depressive symptom detection.

For completeness, we also report results under alternative penalty settings (C=3 and C=3.5) in S6 Text. These supplementary analyses confirm that the methodology remains robust across different cost structures, with combined valence-arousal predictors and discriminant-based models (LDA, QDA) consistently achieving the most balanced performance profiles.

## Discussion

### Principal findings

#### Overview.

The findings from our study demonstrate the potential of our advanced speech analytics combining word sentiment analysis to enhance depressive symptom detection. Our results reveal differences in the valence and arousal scores of words used by family caregivers with low and high depressive symptoms, a population particularly vulnerable to mental health conditions. The preliminary findings highlight the potential of sentimental speech analytics for automated depressive symptom detection. However, it is important to interpret these findings as exploratory and proof-of-concept in nature. This discussion explores the implications of these findings, the advantages of our analytical approach, and the broader significance of utilizing non-invasive methods in mental health assessment, while acknowledging the substantial limitations that must be addressed before clinical application can be considered.

#### Importance of findings.

Depression remains one of the leading mental health conditions globally, affecting millions and contributing to substantial disability [4]. Early depressive symptom detection is crucial in preventing severe consequences, including suicidal ideation [7]. Traditional assessment methods, while effective, often rely on subjective measures that can overlook subtle signs of depression [9–12]. The integration of speech analytics and machine learning presents a novel approach that not only enhances prediction accuracy but also increases the accessibility of mental health services. It may complement existing methods by offering an alternative means of capturing emotional states.

Our study’s findings demonstrate that individuals with high depressive symptoms tend to use language with more negative valence and lower arousal scores compared to those with low depressive symptoms, revealing distinct linguistic patterns that may inform future diagnostic processes. In our empirical analysis, we have demonstrated that the quadratic discriminant analysis (QDA) model with combined valence and arousal predictors achieves a sensitivity of 0.725 and a specificity of 0.563 under repeated 5-fold cross-validation, confirming that these emotional dimensions are useful for classifying levels of depressive symptoms. Similarly, the k-nearest neighbors (kNN) classifier achieved a sensitivity of 0.746 and a specificity of 0.509, further underscoring the utility of these emotional dimensions in distinguishing between depressive symptom groups.

These findings highlight that both valence and arousal scores contribute to prediction, with each dimension shaping classification outcomes in complementary ways. The results demonstrate that emotional signal analysis is not only informative but also clinically relevant, as it enables models to achieve good sensitivity while maintaining reasonable specificity. By leveraging these predictors within a cost-sensitive framework, the methodology reduces false negatives and enhances screening precision, validating valence and arousal analysis as a robust approach for early depressive symptom detection.

#### Advantages of dimensional sentiment analysis.

A key innovation in our study is our use of dimensional sentiment analysis, which evaluates emotional connotations across multiple dimensions—specifically, valence and arousal—using a continuous numerical scale of 1–9. This approach contrasts sharply with traditional categorized sentiment analysis, which often simplifies emotions into binary categories, such as positive or negative and lacks the nuance necessary for understanding the complexities of human emotions [51]. While categorized sentiment analysis can provide a general overview, it lacks the nuance necessary for understanding the complexities of human emotions [52].

Dimensional sentiment analysis allows for a more detailed characterization of emotional states. For example, the valence scale quantifies how positive or negative a word is, while the arousal scale indicates the level of excitement or calmness associated with that word. This multi-dimensional approach is particularly valuable for populations like family caregivers, who may experience a wide range of emotions influenced by their caregiving responsibilities. Our results illustrate that caregivers with high depressive symptoms frequently use words with more extreme valence and arousal ratings, suggesting that their speech contains linguistic markers reflective of their mental state. However, we acknowledge that these findings are preliminary and require replication in larger, more diverse samples.

#### Assessing the influence of outliers on model performance.

As observed in Fig 4, because several observations in the valence and arousal distributions appeared as potential outliers and could influence threshold selection under cost-sensitive learning, we also compare model performance with and without these observations. For a detailed comparison of model performance with and without outliers, readers may refer to S7 Text.

The sensitivity analysis confirms that the main empirical findings are robust: removing outliers produces small numerical adjustments but does not change the qualitative conclusions regarding model performance or the behaviour of cost-sensitive learning.

#### Implications for mental health assessment.

The implications of our findings extend beyond the immediate context of caregiver support. The integration of speech analytics into depressive symptom detection represents a promising step toward more indirect and stigma-sensitive screening methods. Unlike traditional self-report questionnaires, which rely on individuals explicitly endorsing depressive symptoms, our approach analyzes naturalistic speech patterns. This reduces dependence on self-disclosure, which can be influenced by stigma, social desirability bias, or a lack of self-awareness.

*Practical implications*: By focusing on dimensional sentiment analysis, the methodology provides an objective measure of emotional states, offering insights that may not be captured through self-report alone. This is particularly relevant in cultural contexts where discussing mental health remains stigmatized, and individuals may be reluctant to acknowledge or recognize their own symptoms. In such settings, speech-based analysis can serve as a complementary tool, helping to flag potential cases for follow-up without requiring participants to directly confront sensitive questions about their mental health.

We acknowledge that this is not yet a ready-to-deploy clinical tool; rather, our results lay the groundwork for future, larger-scale studies to refine and validate this methodology for potential use in community settings, particularly for populations that may be difficult to reach through conventional mental health services.

*Social implications*: Importantly, this methodology shows potential to circumvent issues around stigma and limited self-awareness during conventional screening processes. By analyzing speech patterns, we may obtain an alternative perspective on an individual’s mental health that is less influenced by social desirability bias. This is especially relevant in cultures where mental health stigma poses a significant barrier to help-seeking. By using open-ended questions about family resilience rather than direct symptom queries, the approach may offer a less threatening entry point for individuals who might otherwise avoid or underreport their distress. Participants are asked to discuss their daily lives, challenges, and sources of strength, rather than being prompted to evaluate their own depressive symptoms. This may yield more spontaneous and authentic emotional expressions that are less filtered by social desirability. Future research should formally evaluate participant acceptability, perceived intrusiveness, and whether the resilience-focused framing indeed mitigates stigma-related response biases.

### Limitations

Several limitations should be considered when interpreting our findings.

*Sample and generalizability*: Our sample comprised 116 family caregivers recruited from a single non-profit organization in Hong Kong. This relatively small and specific sample may limit the generalizability of our findings to other populations, regions, and caregiving contexts. The imbalance between groups (84 low vs. 32 high depressive symptoms) also warrants caution, as performance metrics can be influenced by the majority class. Future research should validate our approach in larger, more diverse samples across different cultural settings.

*Self-report measurement and clinical diagnosis*: While the BDI-II is a well-validated instrument, it relies on self-reported data, which can be subject to bias such as social desirability or limited self-awareness. Importantly, as a formal clinical diagnosis was not ascertained, our findings pertain to elevated depressive symptoms rather than clinically diagnosed depression. Future studies should incorporate structured clinical interviews to establish clinical validity.

*Speech processing and transcription*: The use of automatic speech recognition without manual validation may have introduced transcription errors. Additionally, acoustic features such as tone, pitch, and speech rate, which may carry emotional information, were not incorporated, as our primary focus was on investigating lexical sentiment features. Their exclusion may limit the predictive accuracy of depressive symptoms. Future research could integrate these features and incorporate manual validation to improve data quality and model performance.

*Methodological parameter choices*: While these choices were appropriate for this exploratory study, future research should systematically evaluate how alternative parameter selections affect model robustness and performance.

*Participant acceptability*: We did not systematically collect feedback from participants regarding their comfort level with the interview questions. Thus, the suggestion that resilience-focused questions may be less stigmatizing or intrusive remains speculative and requires empirical validation in future studies.

### Future directions

While our findings provide preliminary evidence for the potential of this approach, substantial further research is needed before clinical application can be considered. First, the efficacy of our approach needs to be validated across diverse populations and contexts. Future studies should explore the applicability of combined dimensional sentiment across different demographic groups, cultural settings, and various mental health conditions. This includes recruiting participants from multiple sites and regions to enhance generalizability. Second, the incorporation of other linguistic features, such as tone, pitch, and speech rate, could enhance the accuracy of depressive symptom detection models. Depression is known to affect not only what is said but also how it is said [20,21]. Integrating acoustic and prosodic features with the lexical features analyzed in this study may improve predictive accuracy. Third, systematic evaluation of participants’ experiences with the interview protocol, including perceived intrusiveness, comfort level, and preferences relative to traditional questionnaires, is essential for assessing the real-world feasibility and ethical acceptability of this approach. Finally, the potential of integrating this technology into digital health platforms presents opportunities for scalable mental health screening, but this should only be pursued after rigorous validation in large, diverse, and clinically characterized samples.

## Conclusion

In conclusion, this study offers proof-of-concept evidence that speech analytics integrating dimensional sentiment analysis can capture linguistic markers associated with depressive symptoms among family caregivers. These findings lay an important foundation for the future development of more accessible and objective screening tools that could complement traditional assessment methods. While substantial refinement and validation in larger, more diverse populations are needed before clinical application can be considered, our results demonstrate the potential of emotional sentiment analysis in speech to inform such efforts. We present this work as a stepping stone toward a more accurate and convenient screening approach, and we encourage future research to build upon these findings by incorporating multi-modal features and evaluating the approach in real-world community settings.

## Supporting information

S1 Text Interview Questions. (DOCX)

S2 Text ASR Transcription, Written Translation, and Annotation Pipeline. (DOCX)

S3 Text Manual Validation of ASR transcription. (DOCX)

S4 Text Manual Validation of Cantonese Verbal Transcript to Written Traditional Chinese Translation. (DOCX)

S5 Text Derivation of the Expected Misclassification Cost Function. (DOCX)

S6 Text Model Performance Results for C = 3 and C = 3.5. (DOCX)

S7 Text Sensitivity Analysis with Outliers Removed. (DOCX)
