# Supplementary material for: Using valence and arousal scoring in speech to detect depressive symptoms: A study on family caregivers
Source: PLoS One. 2026 Oct 14;21(10):e0359556. doi: 10.1371/journal.pone.0359556 (PMC13667775; doi:10.1371/journal.pone.0359556)
Supplement: Supplementary file 1 [file pone.0359556.s001.docx]

**S1 Text.	Interview Questions.**

Table A presents the Interview questions related to Walsh’s family resilience theory.

***Table A***. Interview questions related to Walsh’s family resilience theory

| **Walsh's Theory**  **(Three Broad Process)** | **Interview Questions** |
| --- | --- |
| Family Belief System | 1. What make you stressful? |
|  | 2. When you feel bad, what you will do to make yourself feel better? |
|  | 3. What does it mean by adversity according to your experience? |
|  | 4. What keeps you persevering in the face of adversity? |
| Organizational Pattern | 5. Who is on your mind when you feel weak and why? |
|  | 6. How many new friends did you make at the Centre^#^? |
|  | 7. Do you think making new friends are helpful to you? |
|  | 8. Is there anything make you feel deeply at the Centre^#^? |
| Communicational Pattern | 9. What troubles you the most when taking care of your family members? |
|  | 10. Who do you talk to when you are unhappy and why? |
|  | 11. Who in your family motivates you the most and why? |
|  | 12. What gives you the most satisfaction in caring for your family members? |
| ^#^The non-profit organization provides integrated family and community services in Hong Kong and participated in this study. | |
