# Supplementary material for: Using valence and arousal scoring in speech to detect depressive symptoms: A study on family caregivers
Source: PLoS One. 2026 Oct 14;21(10):e0359556. doi: 10.1371/journal.pone.0359556 (PMC13667775; doi:10.1371/journal.pone.0359556)
Supplement: Supplementary file 2 [file pone.0359556.s002.docx]

**S2 Text***.*	**ASR Transcription, Written Translation, and Annotation Pipeline.**

In this appendix, we present the implementation details of the ASR transcription, written translation, and annotation pipeline.

The end-to-end processing pipeline is organized into three sequential modules:

(1) Audio Pre-processing and **Automatic Speech Recognition (ASR)**

(2) Chunk-based **Spoken-to-Written Translation** from spoken Cantonese to standard Written Chinese

(3) **Affective Annotation** using LLM-derived operators and Chinese Valence–Arousal–Dominance (CVAW) lexicon matching

Each module produces a clearly defined intermediate output that serves as the input to the subsequent module.

## **Module 1: Audio Pre-processing and ASR Transcription**

*Purpose: Convert raw interview audio into clean, sentence-segmented Traditional Chinese transcripts.*

**1.1 Audio Pre-processing and Resampling.** Interview recordings were converted to a standardized single-channel 16 kHz format using the *librosa* library in Python.

**1.2 Automatic Speech Recognition (ASR).** The pre-processed audio was transcribed using the Qwen3-ASR speech-to-text model (Qwen/Qwen3-ASR-1.7B). Language detection was set to automatic mode, enabling the pipeline to handle both Cantonese and English speech within a single workflow.

**1.3 Text Normalization.** Transcribed text was normalized from Simplified to Traditional Chinese characters using the *OpenCC* library with the s2t configuration, ensuring stylistic and linguistic consistency.

**1.4 Sentence Segmentation.** Normalized transcripts were split into discrete sentence segments using regular expressions based on sentence-final punctuation (e.g., 。, ？, ！). This step establishes the sentence boundaries required for subsequent chunking and translation.

*Output of Module 1: Sentence-segmented Traditional Chinese transcripts ready for translation.*

## **Module 2: Chunk-based Written Translation**

*Purpose: Convert spoken-style Cantonese transcripts into standard Written Chinese while preserving meaning and chronological order.*

**2.1 Chunk-Based Parallel Translation.** Sentence segments were grouped into short chunks (5 sentences per unit) and translated concurrently by the qwen3:14b model served via Ollama.

**2.2 Post-Processing and Text Standardization.** Translated outputs were cleaned to remove internal AI reasoning fragments (e.g., <think> tags) and unwanted metadata. Formatting rules were enforced so that all segments conformed to standard Traditional Chinese conventions.

**2.3 Transcript Concatenation.** Cleaned translation segments were merged sequentially in their original chronological order, producing one complete written transcript per interview file.

**2.4 Quality Control and Selective Re-translation.** The concatenated transcripts underwent automated and manual quality checks to identify artifacts, unformatted fragments, or poor translation segments. Problematic segments were selectively re-translated and verified in a secondary review loop, yielding the finalized written Chinese transcripts.

*Output of Module 2: Finalized written Chinese transcripts that serve as the input to the annotation module. We report the manual validation of this translation step in Supplementary Appendix 3.*

## **Module 3: Affective Annotation Pipeline (CVAW and Modifiers)**

*Purpose: Extract modifiers (negation and gradable adverb) and affective words from the finalized written transcripts to support structured analysis of emotional expression.*

**3.1 Initial LLM/CVAW Annotation.** We annotated cleaned transcripts using an LLM-first masking policy across nine modifiers categories (negation, softening negation, strengthening negation, double negation softening, double negation, double negation strengthening, strengthening modifier, softening modifier, and neutral modifier).

**3.2 Tokenization.** We constructed frequency-based templates from the initial LLM/CVAW annotations obtained in Module 3.1. The most frequent LLM-derived labels were transferred onto matching token sequences while preserving CVAW dictionary labels where appropriate.

*Output of Module 3: Token-level affective annotations ready for quantitative analysis.*

The three modules operate sequentially. Module 1 produces the linguistic foundation, Module 2 converts spoken Cantonese style into standard written Chinese, and Module 3 tokenizes the written transcripts. This modular design clarifies data flow and facilitates independent validation of each processing stage.
