# Supplementary material for: Using valence and arousal scoring in speech to detect depressive symptoms: A study on family caregivers
Source: PLoS One. 2026 Oct 14;21(10):e0359556. doi: 10.1371/journal.pone.0359556 (PMC13667775; doi:10.1371/journal.pone.0359556)
Supplement: Supplementary file 3 [file pone.0359556.s003.docx]

**S3 Text***.*	**Manual Validation of ASR transcription**.

**Character Error Rate (CER) for Multilingual ASR Evaluation**

In this section, we present the methodological details and mathematical formulations of the metrics used to measure recognition errors in our multilingual Automatic Speech Recognition (ASR) evaluation.

Researchers increasingly utilize Character Error Rate (CER) as the primary metric for evaluating multilingual ASR systems, particularly for languages without distinct word boundaries. For instance, in character-based languages like Chinese and Cantonese, CER is considered the most appropriate measure since the recognition process operates at a character granularity, tracking more closely with human evaluations than Word Error Rate (WER) [1,2].

This is determined by comparing the recognized text against the original transcription to count the total number of changes required to match them. These changes include substitutions, where a character is incorrect; deletions, where a character is missing; and insertions, where an extra character has been added.

CER is defined as the ratio of the number of character‑level errors to the length of the reference text [2]. Its formula is:

$$\mathrm{CER} = \frac{S + D + I}{N},$$

where $S$, $D$, and $I$ are respectively the numbers of incorrect, missing, and extra characters, and $N$ is the total number of characters in the original transcription.

In addition, we adopt a normalized variant of CER inspired by the Match Error Rate (MER) formulation proposed by Morris et al. [3], where the number of errors is normalized over the total number of edit events plus correct matches. The normalized CER is computed as

$$Normalized CER = \frac{S + D + I}{S + D + I + C},$$

where $C$ is the number of correct characters.

**Manual ASR Transcript Validation and Accuracy**

In this section, we present the baseline metrics and manual validation results for our multilingual ASR transcription accuracy.

On standard Cantonese benchmark datasets, the Whisper-Large baseline yielded an average CER of 21.24%, with results ranging from 19.82% on the low-resource MCE collection to 22.53% on the heavily spontaneous Multi-Domain Cantonese Corpus [4].

We performed the manual validation with six participants (3 participants from the high depressive symptom group and 3 participants from the low depressive symptom group), comparing the machine transcript against manually transcribed, human-verified ground-truth transcripts.

The group-level empirical results are listed in Table B.

***Table B***. Quantitative ASR manual evaluation against human-verified ground-truth transcripts across six participants (three per group), where values for CER and normalized CER are presented as mean (SD).

| **Depressive Symptom Group** | **CER*** | **Normalized CER** | **Min (Normalized CER)** | **Max (Normalized CER)** |
| --- | --- | --- | --- | --- |
| High | 17.78% (1.56%) | 17.29% (1.61%) | 15.45% | 18.39% |
| Low | 19.89% (3.92%) | 19.30% (3.61%) | 15.86% | 23.06% |
| **Overall** | **18.83% (2.91%)** | **18.30% (2.73%)** | **15.45%** | **23.06%** |

* CER is also included for reference.

Across all six participants, the system achieved an overall CER of 18.83% and an overall normalized CER of 18.30%. These results suggest that depressive symptoms and associated acoustic variations have a minimal effect on the dependency features. Consequently, the relatively stable ASR performance across both groups implies that downstream BDI-II score predictions are unlikely to be systematically affected by transcription errors.

**Segmentation Accuracy**

To support the reliability of (a) punctuation and (b) sentence segmentation, we used an FSMN-VAD engine to prevent excessively long audio segments. We then applied an ASR model to automatically insert commas, periods, and question marks, thereby establishing clear sentence boundaries. Text was subsequently split into sentence segments using standard regular expressions based on sentence-final punctuation (e.g., 。, ？, ！). The *PyCantonese* library was utilized for word tokenization (segmentation), as Cantonese lacks spaces between words [5].

We conducted manual spot-checks by randomly auditing a subset of transcripts. The sentence boundaries and word segmentations were generally consistent with human judgment.

**References**

1. K TD, James J, Gopinath DP, Ashraf KM. (2025). Advocating character error rate for multilingual ASR evaluation. In Chiruzzo L, Ritter A, Wang L (Eds.), *Findings of the Association for Computational Linguistics: NAACL 2025* (pp. 4941–4950). Association for Computational Linguistics. <https://doi.org/10.18653/v1/2025.findings-naacl.277>
2. Zhang L, Wu S, Wang Z. (2025). Phoneme-aware hierarchical augmentation and semantic-aware SpecAugment for low-resource Cantonese speech recognition. *Sensors, 25*(14), 4288. <https://doi.org/10.3390/s25144288>
3. Morris AC, Maier V, Green P. (2004). From WER and RIL to MER and WIL: Improved evaluation measures for connected speech recognition. *Proc. Interspeech 2004*, 2765–2768. <https://doi.org/10.21437/Interspeech.2004-668>
4. Chen D, Lin YC, Huang Y, Gong Z, Jiang D, Xie Z, et al. (2025). CantoASR: Prosody-aware ASR-LALM collaboration for low-resource Cantonese. *arXiv preprint arXiv:2511.04139*. <https://doi.org/10.48550/arXiv.2511.04139>
5. Lee JL, Chen L, Lam C, Lau CM, Tsui TH. (2022). PyCantonese: Cantonese linguistics and NLP in python. *In Proceedings of the Thirteenth Language Resources and Evaluation Conference* (pp. 6607–6611). European Language Resources Association. <https://aclanthology.org/2022.lrec-1.711>
