# Supplementary material for: Using valence and arousal scoring in speech to detect depressive symptoms: A study on family caregivers
Source: PLoS One. 2026 Oct 14;21(10):e0359556. doi: 10.1371/journal.pone.0359556 (PMC13667775; doi:10.1371/journal.pone.0359556)
Supplement: Supplementary file 4 [file pone.0359556.s004.docx]

**S4 Text***.*	**Manual Validation of Cantonese Verbal Transcript to Written Traditional Chinese Translation**.

In this section, we present the methodological details and evaluation metrics employed for assessing the quality of converting spoken Cantonese verbal transcripts into standard Written Traditional Chinese. Unlike Automatic Speech Recognition (ASR), where the goal is near-exact character matching against a reference transcription, this task constitutes a form of low-resource style translation. Substantial linguistic divergences exist between spoken Cantonese and standard written Chinese, including lexical substitution, insertion or deletion of modal particles, and systematic word reordering [2]. Consequently, Character Error Rate (CER) alone is inadequate, as many valid and fluent written renderings would be heavily penalized for legitimate structural and lexical differences.

## **Linguistic Considerations in Cantonese-to-Written-Chinese Conversion**

Mak and Lee [2] highlight that spoken Cantonese and standard written Chinese differ not only in vocabulary but also in constituent order. We presented an example illustration in **Table C**.

***Table C*.** A canonical illustration is the request “Give me a cup of water.”

| **English** | Give me a cup of water. |
| --- | --- |
| **Chinese (Written)** | 給我一杯水 (“give” “me” “a” “cup” “water”) |
| **Cantonese (Spoken)** | 畀杯水我 (“give” “cup” “water” “me”) |

Such reorderings, together with lexical mappings (e.g., 畀 → 給) and the systematic removal or rewriting of sentence-final particles, mean that a purely character-level edit-distance metric would conflate genuine translation quality with unavoidable structural divergence. Therefore, we adopt established machine-translation metrics that better accommodate these phenomena.

## **Evaluation Metrics: BLEU and chrF++**

Following common practice in Cantonese–Chinese machine translation research [1], we report BLEU and chrF++.

**BLEU** (Bilingual Evaluation Understudy) measures modified n-gram precision between the system hypothesis and one or more references, combined with a brevity penalty:

$$\mathrm{BLEU}=BP\cdot\exp\left( \sum_{n=1}^{N} w_{n}\log p_{n} \right)$$

where $p_{n}$ are the modified n-gram precision values, $w_{n}$ are uniform weights (typically$N=4$), and $BP$ is the brevity penalty that penalises overly short outputs.

**chrF++** extends the character n-gram F-score (chrF) by additionally incorporating word unigrams and bigrams. It is defined as the harmonic mean of character- and word-level precision and recall over n-grams (character order typically 6, word order 2):

chrF++$=\left( 1+\beta^{2} \right)\cdot\frac{\mathrm{Precision}\cdot\mathrm{Recall}}{\beta^{2}\cdot\mathrm{Precision}+\mathrm{Recall}}$

with $\beta=2$ by default, giving greater weight to recall. Because Chinese and Cantonese are character-based languages without explicit word boundaries, chrF++ is particularly suitable.

## **Reference Performance Ranges**

Lau et al. [1] provide the most directly comparable real-world figures for Cantonese-to-Written-Chinese translation. They partitioned their test set into high-similarity (fewer colloquial features) and low-similarity (more spoken Cantonese features) subsets.

The low-similarity case is the more realistic reference for systems operating on raw spoken transcripts. Scores in the vicinity of BLEU ≈ 30 and chrF++ ≈ 22 to 23 therefore represent competitive performance for this difficult, low-resource conversion task. We presented a summary of the evaluation results in **Table D**.

## **Table D. Summary of Evaluation Results**

| **System / Condition (Case)** | **BLEU** | **chrF++** | **Remark** |
| --- | --- | --- | --- |
| Lau et al. [1] – High-similarity | 64.3 | 44.8 | Easier subset |
| Lau et al. [1] – Low-similarity | 30.1 | 22.6 | More spoken features |
| Our system (3 BDI-High + 3 BDI-Low = 6 representative cases) | 24.4–35.3 | 18.9–27.6 | Spoken → Written Chinese |
| Our system (Mean of the six representative cases) | 30.20 | 23.58 |  |

These automatic metrics, supplemented by qualitative inspection of lexical choice and word-order fidelity, constitute the primary validation framework for the Cantonese verbal-to-Written-Traditional-Chinese translation component.

**References**

1. Mak HY, Lee T. (2022). Low-Resource NMT: A Case Study on the Written and Spoken Languages in Hong Kong. *In Proceedings of the 2021 5th International Conference on Natural Language Processing and Information Retrieval* (NLPIR '21) (pp. 81–87). Association for Computing Machinery. <https://doi.org/10.1145/3508230.3508242>
2. Lau LL, Tsang TY, Ng MK, Kwok RY, Au Yeung SK. (2025). Real-World Application of Machine Translation of Cantonese-to-Written-Chinese Translation. *In Proceedings of the 2024 8th International Conference on Natural Language Processing and Information Retrieval* (NLPIR 2024) (pp. 199–205). Association for Computing Machinery. <https://doi.org/10.1145/3711542.3711546>
