# Supplementary material for: Using valence and arousal scoring in speech to detect depressive symptoms: A study on family caregivers
Source: PLoS One. 2026 Oct 14;21(10):e0359556. doi: 10.1371/journal.pone.0359556 (PMC13667775; doi:10.1371/journal.pone.0359556)
Supplement: Supplementary file 5 [file pone.0359556.s005.docx]

**S5 Text***.*	**Derivation of the Expected Misclassification Cost Function**.

To formalize the expected misclassification cost in binary classification, we begin by defining the key performance measures. Let $y\in\{0,1\}$ denote the true class label of an individual, where $y=1$ indicates a positive case (high depressive symptom) and $y=0$ a negative case (low depressive symptom). Let $\hat{y}\in\{0,1\}$ denote the predicted label produced by the classifier. Sensitivity is the probability of correctly identifying a positive case, $\text{Sensitivity}=P(\hat{y}=1\mid y=1)$. Its complement, $1-\text{Sensitivity}$, represents the false negative rate, i.e., the probability of misclassifying a true positive as negative. Specificity is the probability of correctly identifying a negative case, $\text{Specificity}=P(\hat{y}=0\mid y=0)$. Its complement, $1-\text{Specificity}$, represents the false positive rate, i.e., the probability of misclassifying a true negative as positive.

Let $\pi_{1}=P(y=1)$ denote the prevalence of positive cases in the population, and $\pi_{0}=P\left( y=0 \right)\equiv1-\pi_{1}$ the prevalence of negative cases. These prevalence values can be interpreted as the prior probabilities of observing a positive or negative case before prediction. Finally, let $C$ denote the relative cost of a false negative compared to a false positive, with the latter normalized to have cost equal to one.

The cost function can be expressed using indicator functions for misclassification events:

$$\text{Cost}(y,\hat{y})=C\cdot\mathbf{1}\{y=1,\hat{y}=0\}\text{ }+\text{ }\mathbf{1}\{y=0,\hat{y}=1\},$$

where $\mathbf{1}\{\cdot\}$ is the indicator function that equals 1 if the condition is true and 0 otherwise.

Taking expectations over the joint distribution of $\left( y , \hat{y} \right)$, this reduces to:

| $\mathbb{E}\left[ \text{Cost}\left( y,\hat{y} \right) \right]$ | $=C\cdot P\left( y=1,\hat{y}=0 \right)+P\left( y=0,\hat{y}=1 \right)$ |  |
| --- | --- | --- |
|  | $=C\cdot P\left( \hat{y}=0\vert y=1 \right)P\left( y=1 \right)+P\left( \hat{y}=1\vert y=0 \right)p\left( y=0 \right)$ |  |
|  | $=C\cdot\left( 1-\mathrm{Sensitivity} \right)\pi_{1}+\left( 1-\mathrm{Specificity} \right)\pi_{0}$ |  |

Thus, the expected cost function is derived directly from the definition of misclassification events: false negatives contribute a penalty weighted by $C$ and the prior probability of positives, while false positives contribute a penalty weighted by the prior probability of negatives.

In application of early depressive symptom detection, it is appropriate to set $C>1$. A false negative in this setting corresponds to failing to identify an individual who has high depressive symptoms, which can delay treatment and increase the risk of severe outcomes. By contrast, a false positive only results in additional screening or evaluation, which carries comparatively minor consequences. Assigning a higher relative cost to false negatives ensures that the expected cost function penalizes missed cases more heavily than unnecessary alerts. This weighting shifts the evaluation toward prioritizing sensitivity, aligning the model’s performance with the ethical and clinical imperative to minimize undetected depressive symptoms.
