# Supplementary material for: Using valence and arousal scoring in speech to detect depressive symptoms: A study on family caregivers
Source: PLoS One. 2026 Oct 14;21(10):e0359556. doi: 10.1371/journal.pone.0359556 (PMC13667775; doi:10.1371/journal.pone.0359556)
Supplement: Supplementary file 6 [file pone.0359556.s006.docx]

**S6 Text***.*	**Model Performance Results for** $C=3$ **and** $C=3.5$.

Tables E and F present the model performance results for $C=3$ and $C=3.5$. Among the single‑predictor models, valence‑based classifiers achieved moderate sensitivity with relatively balanced specificity. For example, under $C=3$, QDA reached a sensitivity of 0.691 and a specificity of 0.593, while LDA achieved 0.616 sensitivity and 0.642 specificity. At $C=3.5$, sensitivity improved further (QDA = 0.728, LDA = 0.646), though specificity declined slightly (QDA = 0.556, LDA = 0.602). These results suggest that valence features alone provide a stable baseline, but their discriminative power is enhanced when the penalty for false negatives is increased.

By contrast, arousal‑based models tended to yield higher sensitivity but at the expense of specificity. Under $C=3$, LDA achieved a sensitivity of 0.466 and a specificity of 0.693, while QDA produced 0.457 sensitivity and 0.693 specificity. At $C=3.5$, sensitivity rose (LDA = 0.591, QDA = 0.591), but specificity remained modest (LDA = 0.510, QDA = 0.497). This pattern indicates that arousal features capture positive cases more aggressively, but with limited precision in distinguishing negatives.

When valence and arousal predictors were combined, performance improved across several metrics. Sensitivity remained robust while specificity increased, producing more balanced outcomes. Under $C=3$, QDA achieved a sensitivity of 0.670 and a specificity of 0.630, while kNN reached 0.668 sensitivity and 0.600 specificity. At $C=3.5$, QDA improved to 0.703 sensitivity and 0.591 specificity, and kNN achieved 0.713 sensitivity and 0.551 specificity. This underscores the value of integrating both emotional dimensions.

These findings confirm that the framework adapts effectively to different cost structures, maintaining reliable classification performance while aligning with the ethical imperative of minimizing undetected depression. The robustness of results across multiple penalty values underscores the methodological strength of cost‑sensitive cutoff selection and validates its application in early depressive symptom detection.

**Table E.** Average performance metrics and 95% credible intervals (CIs) for nine machine learning classifiers under repeated 5‑fold nested cross‑validation with 100 independent repetitions. Models were evaluated using three predictor configurations: (1) valence‑related statistics alone, (2) arousal‑related statistics alone, and (3) combined valence and arousal statistics. Reported metrics include sensitivity, specificity, expected cost, AUC, accuracy, positive predictive value (PPV), negative predictive value (NPV), and F1‑score. Within each repetition, the inner CV‑folds were used to select the classification cutoff by minimizing the expected cost function, thereby adapting the threshold to balance sensitivity and specificity under asymmetric misclassification costs. A cost‑sensitive penalty of $C=3$ was applied, reflecting the higher clinical importance of avoiding false negatives in early depressive symptom detection.

| Model | Predictor | Sensitivity | Specificity | Expected cost | AUC | Accuracy | PPV | NPV | F1 score |
| --- | --- | --- | --- | --- | --- | --- | --- | --- | --- |
| Mode1 1: Logistic regression | Valence | 0.607 (0.31, 0.842) | 0.54 (0.204, 0.83) | 1.652 (1.152, 2.261) | 0.67 (0.605, 0.708) | 0.559 (0.389, 0.699) | 0.324 (0.198, 0.472) | 0.633 (0.311, 0.835) | 0.394 (0.25, 0.51) |
| Mode1 2: Random Forest |  | 0.698 (0.567, 0.852) | 0.509 (0.389, 0.622) | 1.388 (0.981, 1.735) | 0.639 (0.564, 0.717) | 0.562 (0.491, 0.635) | 0.356 (0.294, 0.429) | 0.828 (0.686, 0.906) | 0.459 (0.385, 0.535) |
| Mode1 3: MLP classifier |  | 0.714 (0.539, 0.871) | 0.476 (0.312, 0.594) | 1.377 (1.001, 1.816) | 0.648 (0.56, 0.73) | 0.542 (0.464, 0.63) | 0.348 (0.28, 0.432) | 0.823 (0.64, 0.913) | 0.456 (0.38, 0.542) |
| Mode1 4: XGB classifier |  | 0.679 (0.505, 0.814) | 0.497 (0.382, 0.608) | 1.454 (1.024, 1.883) | 0.632 (0.549, 0.698) | 0.547 (0.468, 0.626) | 0.345 (0.263, 0.435) | 0.82 (0.718, 0.908) | 0.442 (0.349, 0.518) |
| Mode1 5: Gradient boosting classifier |  | 0.701 (0.466, 0.87) | 0.436 (0.278, 0.61) | 1.452 (1.051, 1.907) | 0.604 (0.503, 0.684) | 0.509 (0.42, 0.586) | 0.325 (0.247, 0.383) | 0.793 (0.604, 0.895) | 0.43 (0.326, 0.492) |
| Mode1 6: Support vector machine |  | 0.587 (0.31, 0.833) | 0.503 (0.247, 0.746) | 1.743 (1.197, 2.412) | 0.584 (0.418, 0.691) | 0.527 (0.391, 0.654) | 0.321 (0.199, 0.458) | 0.658 (0.424, 0.868) | 0.381 (0.239, 0.481) |
| Mode1 7: kNN |  | 0.698 (0.549, 0.847) | 0.56 (0.426, 0.67) | 1.352 (0.971, 1.742) | 0.681 (0.624, 0.74) | 0.598 (0.518, 0.665) | 0.386 (0.316, 0.455) | 0.85 (0.787, 0.918) | 0.48 (0.396, 0.548) |
| Mode1 8: LDA |  | 0.616 (0.529, 0.713) | 0.642 (0.548, 0.715) | 1.506 (1.264, 1.792) | 0.682 (0.641, 0.711) | 0.635 (0.576, 0.682) | 0.41 (0.311, 0.489) | 0.824 (0.789, 0.858) | 0.475 (0.391, 0.542) |
| Mode1 9: QDA |  | 0.691 (0.572, 0.812) | 0.593 (0.48, 0.679) | 1.336 (0.985, 1.642) | 0.7 (0.648, 0.738) | 0.62 (0.545, 0.674) | 0.4 (0.313, 0.46) | 0.847 (0.806, 0.892) | 0.493 (0.425, 0.565) |
| Mode1 1: Logistic regression | Arousal | 0.586 (0.294, 0.863) | 0.501 (0.189, 0.829) | 1.74 (1.166, 2.348) | 0.637 (0.593, 0.665) | 0.526 (0.362, 0.68) | 0.31 (0.17, 0.448) | 0.526 (0.187, 0.8) | 0.361 (0.216, 0.466) |
| Mode1 2: Random Forest |  | 0.554 (0.35, 0.718) | 0.503 (0.276, 0.689) | 1.846 (1.455, 2.346) | 0.567 (0.498, 0.627) | 0.517 (0.389, 0.635) | 0.303 (0.22, 0.405) | 0.693 (0.432, 0.824) | 0.371 (0.261, 0.452) |
| Mode1 3: MLP classifier |  | 0.467 (0.305, 0.666) | 0.617 (0.373, 0.822) | 1.979 (1.508, 2.392) | 0.58 (0.494, 0.65) | 0.575 (0.439, 0.698) | 0.347 (0.228, 0.523) | 0.728 (0.541, 0.82) | 0.36 (0.254, 0.456) |
| Mode1 4: XGB classifier |  | 0.519 (0.33, 0.694) | 0.533 (0.313, 0.715) | 1.908 (1.475, 2.387) | 0.56 (0.489, 0.633) | 0.529 (0.406, 0.644) | 0.303 (0.208, 0.435) | 0.71 (0.522, 0.815) | 0.36 (0.266, 0.452) |
| Mode1 5: Gradient boosting classifier |  | 0.584 (0.334, 0.798) | 0.421 (0.168, 0.661) | 1.822 (1.366, 2.357) | 0.529 (0.454, 0.61) | 0.465 (0.335, 0.602) | 0.275 (0.187, 0.356) | 0.577 (0.342, 0.782) | 0.351 (0.237, 0.427) |
| Mode1 6: Support vector machine |  | 0.61 (0.298, 0.833) | 0.409 (0.153, 0.727) | 1.779 (1.324, 2.42) | 0.487 (0.361, 0.623) | 0.464 (0.32, 0.621) | 0.288 (0.158, 0.49) | 0.405 (0.15, 0.671) | 0.338 (0.197, 0.444) |
| Mode1 7: kNN |  | 0.491 (0.339, 0.641) | 0.655 (0.464, 0.81) | 1.863 (1.528, 2.231) | 0.601 (0.535, 0.65) | 0.611 (0.503, 0.698) | 0.383 (0.263, 0.518) | 0.758 (0.597, 0.831) | 0.396 (0.312, 0.488) |
| Mode1 8: LDA |  | 0.466 (0.367, 0.598) | 0.693 (0.496, 0.821) | 1.906 (1.62, 2.112) | 0.649 (0.623, 0.671) | 0.63 (0.529, 0.714) | 0.402 (0.282, 0.536) | 0.773 (0.633, 0.813) | 0.397 (0.322, 0.473) |
| Mode1 9: QDA |  | 0.457 (0.348, 0.594) | 0.693 (0.515, 0.844) | 1.934 (1.599, 2.168) | 0.642 (0.597, 0.676) | 0.627 (0.521, 0.724) | 0.403 (0.274, 0.542) | 0.764 (0.611, 0.813) | 0.39 (0.304, 0.464) |
| Mode1 1: Logistic regression | Valence and arousal | 0.624 (0.392, 0.813) | 0.604 (0.359, 0.765) | 1.533 (1.091, 2.043) | 0.669 (0.616, 0.71) | 0.61 (0.457, 0.69) | 0.389 (0.245, 0.514) | 0.76 (0.465, 0.865) | 0.451 (0.319, 0.545) |
| Mode1 2: Random Forest |  | 0.656 (0.496, 0.808) | 0.535 (0.421, 0.678) | 1.506 (1.111, 1.888) | 0.648 (0.583, 0.714) | 0.569 (0.484, 0.663) | 0.362 (0.292, 0.448) | 0.812 (0.676, 0.884) | 0.448 (0.358, 0.522) |
| Mode1 3: MLP classifier |  | 0.598 (0.443, 0.775) | 0.49 (0.319, 0.639) | 1.729 (1.345, 2.144) | 0.587 (0.504, 0.66) | 0.52 (0.432, 0.613) | 0.317 (0.22, 0.439) | 0.748 (0.586, 0.837) | 0.395 (0.298, 0.47) |
| Mode1 4: XGB classifier |  | 0.637 (0.478, 0.779) | 0.587 (0.434, 0.723) | 1.502 (1.198, 1.892) | 0.654 (0.583, 0.732) | 0.601 (0.524, 0.68) | 0.382 (0.294, 0.503) | 0.819 (0.742, 0.879) | 0.459 (0.357, 0.552) |
| Mode1 5: Gradient boosting classifier |  | 0.674 (0.496, 0.87) | 0.489 (0.29, 0.686) | 1.498 (1.107, 1.931) | 0.628 (0.559, 0.715) | 0.54 (0.439, 0.646) | 0.345 (0.273, 0.43) | 0.774 (0.518, 0.883) | 0.44 (0.341, 0.511) |
| Mode1 6: Support vector machine |  | 0.632 (0.378, 0.87) | 0.45 (0.157, 0.736) | 1.664 (1.104, 2.248) | 0.588 (0.424, 0.714) | 0.5 (0.347, 0.663) | 0.33 (0.233, 0.494) | 0.558 (0.294, 0.796) | 0.393 (0.304, 0.499) |
| Mode1 7: kNN |  | 0.668 (0.524, 0.833) | 0.6 (0.488, 0.714) | 1.398 (1.014, 1.779) | 0.692 (0.642, 0.736) | 0.619 (0.536, 0.673) | 0.4 (0.317, 0.477) | 0.841 (0.786, 0.901) | 0.483 (0.387, 0.555) |
| Mode1 8: LDA |  | 0.646 (0.533, 0.723) | 0.656 (0.571, 0.737) | 1.405 (1.172, 1.694) | 0.685 (0.652, 0.712) | 0.653 (0.594, 0.714) | 0.428 (0.346, 0.506) | 0.837 (0.794, 0.868) | 0.501 (0.424, 0.568) |
| Mode1 9: QDA |  | 0.67 (0.543, 0.814) | 0.63 (0.524, 0.728) | 1.364 (0.98, 1.704) | 0.696 (0.643, 0.737) | 0.641 (0.57, 0.699) | 0.421 (0.338, 0.511) | 0.842 (0.793, 0.899) | 0.502 (0.426, 0.571) |

**Table F.** Average performance metrics and 95% credible intervals (CIs) for nine machine learning classifiers under repeated 5‑fold nested cross‑validation with 100 independent repetitions. The metrics are the same as Table E, but with a penalty of $C=3.5$.

| Model | Predictor | Sensitivity | Specificity | Expected cost | AUC | Accuracy | PPV | NPV | F1 score |
| --- | --- | --- | --- | --- | --- | --- | --- | --- | --- |
| Mode1 1: Logistic regression | Valence | 0.717 (0.473, 0.898) | 0.423 (0.189, 0.703) | 1.574 (1.143, 2.214) | 0.67 (0.605, 0.708) | 0.504 (0.372, 0.647) | 0.334 (0.238, 0.469) | 0.568 (0.31, 0.839) | 0.43 (0.31, 0.518) |
| Mode1 2: Random Forest |  | 0.751 (0.61, 0.871) | 0.442 (0.296, 0.58) | 1.426 (1.048, 1.83) | 0.639 (0.564, 0.717) | 0.528 (0.432, 0.613) | 0.346 (0.297, 0.409) | 0.826 (0.644, 0.912) | 0.463 (0.4, 0.533) |
| Mode1 3: MLP classifier |  | 0.769 (0.587, 0.933) | 0.42 (0.263, 0.548) | 1.378 (0.9, 1.943) | 0.648 (0.56, 0.73) | 0.516 (0.424, 0.604) | 0.341 (0.281, 0.412) | 0.825 (0.632, 0.942) | 0.463 (0.382, 0.539) |
| Mode1 4: XGB classifier |  | 0.724 (0.559, 0.876) | 0.451 (0.332, 0.548) | 1.514 (1.005, 1.991) | 0.632 (0.549, 0.698) | 0.526 (0.456, 0.593) | 0.34 (0.278, 0.399) | 0.818 (0.656, 0.912) | 0.45 (0.36, 0.526) |
| Mode1 5: Gradient boosting classifier |  | 0.759 (0.591, 0.898) | 0.37 (0.189, 0.526) | 1.47 (1.099, 1.912) | 0.604 (0.503, 0.684) | 0.477 (0.378, 0.56) | 0.32 (0.261, 0.374) | 0.772 (0.543, 0.914) | 0.438 (0.366, 0.491) |
| Mode1 6: Support vector machine |  | 0.682 (0.426, 0.904) | 0.391 (0.154, 0.648) | 1.737 (1.152, 2.34) | 0.584 (0.418, 0.691) | 0.472 (0.346, 0.595) | 0.31 (0.215, 0.427) | 0.592 (0.306, 0.871) | 0.399 (0.293, 0.478) |
| Mode1 7: kNN |  | 0.75 (0.591, 0.909) | 0.511 (0.393, 0.607) | 1.368 (0.927, 1.843) | 0.681 (0.624, 0.74) | 0.577 (0.508, 0.636) | 0.378 (0.313, 0.434) | 0.866 (0.791, 0.931) | 0.488 (0.409, 0.553) |
| Mode1 8: LDA |  | 0.646 (0.539, 0.752) | 0.602 (0.511, 0.691) | 1.629 (1.294, 1.951) | 0.682 (0.641, 0.711) | 0.614 (0.538, 0.665) | 0.393 (0.303, 0.472) | 0.829 (0.783, 0.869) | 0.473 (0.392, 0.542) |
| Mode1 9: QDA |  | 0.728 (0.615, 0.823) | 0.556 (0.45, 0.654) | 1.394 (1.075, 1.773) | 0.7 (0.648, 0.738) | 0.603 (0.535, 0.67) | 0.392 (0.307, 0.459) | 0.857 (0.813, 0.905) | 0.497 (0.421, 0.555) |
| Mode1 1: Logistic regression | Arousal | 0.751 (0.534, 0.997) | 0.304 (0.029, 0.568) | 1.567 (1, 2.058) | 0.637 (0.593, 0.665) | 0.427 (0.293, 0.559) | 0.303 (0.221, 0.439) | 0.409 (0.129, 0.693) | 0.401 (0.315, 0.471) |
| Mode1 2: Random Forest |  | 0.674 (0.491, 0.819) | 0.347 (0.153, 0.576) | 1.784 (1.407, 2.248) | 0.567 (0.498, 0.627) | 0.437 (0.33, 0.567) | 0.286 (0.221, 0.363) | 0.573 (0.275, 0.828) | 0.385 (0.299, 0.442) |
| Mode1 3: MLP classifier |  | 0.617 (0.397, 0.809) | 0.415 (0.202, 0.647) | 1.926 (1.398, 2.548) | 0.58 (0.494, 0.65) | 0.471 (0.36, 0.587) | 0.296 (0.218, 0.369) | 0.642 (0.363, 0.821) | 0.376 (0.268, 0.446) |
| Mode1 4: XGB classifier |  | 0.636 (0.462, 0.799) | 0.389 (0.178, 0.575) | 1.89 (1.426, 2.285) | 0.56 (0.489, 0.633) | 0.457 (0.345, 0.551) | 0.287 (0.219, 0.348) | 0.625 (0.366, 0.809) | 0.377 (0.311, 0.444) |
| Mode1 5: Gradient boosting classifier |  | 0.734 (0.524, 0.967) | 0.251 (0.024, 0.468) | 1.683 (1.084, 2.316) | 0.529 (0.454, 0.61) | 0.384 (0.276, 0.493) | 0.268 (0.197, 0.335) | 0.401 (0.103, 0.716) | 0.378 (0.284, 0.447) |
| Mode1 6: Support vector machine |  | 0.763 (0.491, 1) | 0.231 (0, 0.514) | 1.614 (1, 2.32) | 0.487 (0.361, 0.623) | 0.378 (0.276, 0.532) | 0.274 (0.202, 0.41) | 0.287 (0, 0.552) | 0.376 (0.275, 0.441) |
| Mode1 7: kNN |  | 0.593 (0.419, 0.757) | 0.509 (0.303, 0.725) | 1.906 (1.549, 2.391) | 0.601 (0.535, 0.65) | 0.532 (0.405, 0.654) | 0.341 (0.259, 0.455) | 0.655 (0.434, 0.849) | 0.399 (0.316, 0.496) |
| Mode1 8: LDA |  | 0.591 (0.438, 0.752) | 0.51 (0.272, 0.677) | 1.916 (1.521, 2.325) | 0.649 (0.623, 0.671) | 0.532 (0.382, 0.645) | 0.336 (0.244, 0.469) | 0.745 (0.519, 0.837) | 0.397 (0.321, 0.474) |
| Mode1 9: QDA |  | 0.591 (0.415, 0.776) | 0.497 (0.291, 0.726) | 1.925 (1.466, 2.355) | 0.642 (0.597, 0.676) | 0.523 (0.406, 0.647) | 0.332 (0.233, 0.436) | 0.736 (0.526, 0.835) | 0.391 (0.319, 0.454) |
| Mode1 1: Logistic regression | Valence and arousal | 0.702 (0.543, 0.871) | 0.519 (0.299, 0.69) | 1.532 (1.063, 2.079) | 0.669 (0.616, 0.71) | 0.571 (0.426, 0.673) | 0.382 (0.279, 0.478) | 0.728 (0.452, 0.872) | 0.472 (0.363, 0.547) |
| Mode1 2: Random Forest |  | 0.716 (0.562, 0.848) | 0.459 (0.299, 0.616) | 1.53 (1.178, 2.001) | 0.648 (0.583, 0.714) | 0.529 (0.441, 0.621) | 0.344 (0.288, 0.424) | 0.816 (0.63, 0.894) | 0.45 (0.376, 0.522) |
| Mode1 3: MLP classifier |  | 0.685 (0.501, 0.823) | 0.383 (0.227, 0.571) | 1.727 (1.305, 2.264) | 0.587 (0.504, 0.66) | 0.466 (0.378, 0.577) | 0.301 (0.228, 0.375) | 0.723 (0.486, 0.865) | 0.406 (0.308, 0.484) |
| Mode1 4: XGB classifier |  | 0.686 (0.524, 0.81) | 0.532 (0.355, 0.677) | 1.575 (1.207, 2.067) | 0.654 (0.583, 0.732) | 0.575 (0.476, 0.655) | 0.371 (0.272, 0.448) | 0.821 (0.646, 0.89) | 0.466 (0.362, 0.54) |
| Mode1 5: Gradient boosting classifier |  | 0.745 (0.581, 0.909) | 0.391 (0.2, 0.568) | 1.486 (1.005, 1.912) | 0.628 (0.559, 0.715) | 0.488 (0.373, 0.602) | 0.327 (0.266, 0.39) | 0.72 (0.436, 0.887) | 0.442 (0.372, 0.501) |
| Mode1 6: Support vector machine |  | 0.712 (0.487, 0.914) | 0.355 (0.118, 0.667) | 1.655 (1.065, 2.188) | 0.588 (0.424, 0.714) | 0.454 (0.328, 0.631) | 0.32 (0.24, 0.457) | 0.494 (0.205, 0.819) | 0.407 (0.319, 0.483) |
| Mode1 7: kNN |  | 0.713 (0.586, 0.838) | 0.551 (0.44, 0.666) | 1.45 (1.063, 1.874) | 0.692 (0.642, 0.736) | 0.596 (0.526, 0.664) | 0.389 (0.317, 0.461) | 0.85 (0.785, 0.912) | 0.488 (0.423, 0.559) |
| Mode1 8: LDA |  | 0.668 (0.571, 0.752) | 0.628 (0.538, 0.715) | 1.529 (1.284, 1.867) | 0.685 (0.652, 0.712) | 0.639 (0.579, 0.69) | 0.418 (0.337, 0.487) | 0.841 (0.799, 0.878) | 0.501 (0.418, 0.562) |
| Mode1 9: QDA |  | 0.703 (0.581, 0.819) | 0.591 (0.43, 0.699) | 1.447 (1.058, 1.889) | 0.696 (0.643, 0.737) | 0.622 (0.518, 0.697) | 0.408 (0.325, 0.479) | 0.844 (0.762, 0.9) | 0.503 (0.41, 0.57) |
