# Supplementary material for: Using valence and arousal scoring in speech to detect depressive symptoms: A study on family caregivers
Source: PLoS One. 2026 Oct 14;21(10):e0359556. doi: 10.1371/journal.pone.0359556 (PMC13667775; doi:10.1371/journal.pone.0359556)
Supplement: Supplementary file 7 [file pone.0359556.s007.docx]

**S7 Text***.*	**Sensitivity Analysis with Outliers Removed**.

This sensitivity analysis examined whether extreme observations in the valence and arousal distributions influenced the performance of the machine learning classifiers under cost‑sensitive threshold selection. As observed in Figure 4 of the main text, there are some outliers in the mean valence and arousal scores, which could potentially affect the performances of the machine learning models. To evaluate this possibility, all models were re‑estimated after removing outliers, and their performance was compared against the original results under penalties C = 3, 3.5, and 4 in respectively Tables G, H, and I.

Across all penalty levels, the comparison shows that **the effect of outlier removal is mild**, and the overall performance patterns remain highly consistent. The ranking of models, the relative strength of valence versus arousal predictors, and the behaviour of cost‑sensitive thresholding all remain essentially unchanged.

Several examples illustrate this stability:

- Under **C = 3**, logistic regression using valence predictors originally had sensitivity 0.607 and specificity 0.540. After outlier removal, these became 0.581 and 0.583, respectively.
- For **combined valence-arousal predictors** under C = 3, kNN originally had sensitivity 0.668 and specificity 0.600. With outliers removed, the values were 0.678 and 0.606.
- Under **C = 3.5,** random forest with valence predictors showed sensitivity 0.751 and specificity 0.442 in the original analysis, compared with 0.734 and 0.459 after outlier removal.
- Under **C = 4**, QDA with combined predictors originally had sensitivity 0.725 and specificity 0.563. After removing outliers, the values were 0.740 and 0.572.

These differences are numerically minor and do not change the models’ profiles. The credible intervals from the original and outlier‑removed analyses overlap substantially, indicating that the variability introduced by repeated nested cross‑validation dominates any influence of outliers. The combined valence–arousal predictors continue to provide the strongest overall balance of sensitivity and specificity. The sensitivity analysis therefore confirms that the main empirical findings are robust: removing outliers produces small numerical adjustments but **does not change the qualitative conclusions** regarding model performance or the behaviour of cost‑sensitive learning.

**Table G.** Average performance metrics and 95% credible intervals (CIs) for nine machine learning classifiers under repeated 5‑fold nested cross‑validation with 100 independent repetitions with a penalty of $C=3$, with outliers removed.

| Model | Predictor | Sensitivity | Specificity | Expected cost | AUC | Accuracy | PPV | NPV | F1 score |
| --- | --- | --- | --- | --- | --- | --- | --- | --- | --- |
| Mode1 1: Logistic regression | Valence | 0.581 (0.309, 0.81) | 0.583 (0.282, 0.814) | 1.674 (1.187, 2.283) | 0.68 (0.629, 0.713) | 0.587 (0.425, 0.711) | 0.315 (0.194, 0.438) | 0.715 (0.451, 0.86) | 0.391 (0.255, 0.511) |
| Mode1 2: Random Forest |  | 0.684 (0.535, 0.814) | 0.518 (0.381, 0.652) | 1.43 (1.124, 1.795) | 0.637 (0.575, 0.692) | 0.565 (0.495, 0.632) | 0.362 (0.304, 0.42) | 0.817 (0.702, 0.882) | 0.462 (0.375, 0.52) |
| Mode1 3: MLP classifier |  | 0.705 (0.529, 0.865) | 0.479 (0.304, 0.619) | 1.406 (1, 1.869) | 0.647 (0.566, 0.713) | 0.542 (0.434, 0.631) | 0.353 (0.293, 0.426) | 0.808 (0.634, 0.918) | 0.455 (0.371, 0.536) |
| Mode1 4: XGB classifier |  | 0.676 (0.507, 0.845) | 0.522 (0.374, 0.69) | 1.451 (1.054, 1.815) | 0.643 (0.57, 0.716) | 0.565 (0.483, 0.654) | 0.362 (0.293, 0.45) | 0.82 (0.728, 0.889) | 0.455 (0.366, 0.535) |
| Mode1 5: Gradient boosting classifier |  | 0.726 (0.552, 0.876) | 0.425 (0.256, 0.598) | 1.395 (1.026, 1.858) | 0.612 (0.556, 0.672) | 0.51 (0.411, 0.589) | 0.337 (0.274, 0.392) | 0.78 (0.537, 0.891) | 0.448 (0.37, 0.512) |
| Mode1 6: Support vector machine |  | 0.61 (0.336, 0.874) | 0.477 (0.152, 0.732) | 1.693 (1.181, 2.319) | 0.591 (0.454, 0.684) | 0.515 (0.35, 0.649) | 0.322 (0.205, 0.458) | 0.667 (0.39, 0.835) | 0.391 (0.258, 0.5) |
| Mode1 7: kNN |  | 0.707 (0.543, 0.848) | 0.561 (0.429, 0.667) | 1.317 (0.905, 1.771) | 0.677 (0.618, 0.736) | 0.603 (0.532, 0.672) | 0.399 (0.341, 0.481) | 0.85 (0.777, 0.913) | 0.495 (0.412, 0.572) |
| Mode1 8: LDA |  | 0.635 (0.547, 0.726) | 0.64 (0.529, 0.725) | 1.456 (1.246, 1.698) | 0.691 (0.658, 0.717) | 0.639 (0.579, 0.702) | 0.423 (0.358, 0.505) | 0.825 (0.791, 0.852) | 0.492 (0.43, 0.547) |
| Mode1 9: QDA |  | 0.702 (0.569, 0.814) | 0.6 (0.5, 0.695) | 1.295 (1.02, 1.664) | 0.707 (0.662, 0.739) | 0.628 (0.575, 0.684) | 0.416 (0.331, 0.468) | 0.849 (0.806, 0.9) | 0.508 (0.421, 0.564) |
| Mode1 1: Logistic regression | Arousal | 0.568 (0.269, 0.85) | 0.514 (0.214, 0.78) | 1.783 (1.173, 2.467) | 0.642 (0.601, 0.671) | 0.53 (0.382, 0.65) | 0.307 (0.145, 0.441) | 0.542 (0.28, 0.798) | 0.352 (0.195, 0.459) |
| Mode1 2: Random Forest |  | 0.556 (0.39, 0.767) | 0.511 (0.32, 0.682) | 1.82 (1.334, 2.219) | 0.573 (0.49, 0.631) | 0.523 (0.416, 0.622) | 0.312 (0.224, 0.383) | 0.699 (0.531, 0.812) | 0.378 (0.283, 0.458) |
| Mode1 3: MLP classifier |  | 0.499 (0.34, 0.683) | 0.58 (0.368, 0.766) | 1.924 (1.544, 2.247) | 0.586 (0.502, 0.663) | 0.557 (0.438, 0.672) | 0.341 (0.235, 0.473) | 0.715 (0.544, 0.801) | 0.373 (0.286, 0.442) |
| Mode1 4: XGB classifier |  | 0.547 (0.345, 0.748) | 0.519 (0.319, 0.709) | 1.84 (1.358, 2.321) | 0.567 (0.467, 0.642) | 0.527 (0.42, 0.632) | 0.318 (0.23, 0.418) | 0.711 (0.558, 0.816) | 0.378 (0.265, 0.473) |
| Mode1 5: Gradient boosting classifier |  | 0.604 (0.41, 0.82) | 0.415 (0.174, 0.61) | 1.773 (1.288, 2.252) | 0.544 (0.457, 0.625) | 0.469 (0.352, 0.587) | 0.291 (0.202, 0.371) | 0.567 (0.289, 0.808) | 0.366 (0.26, 0.452) |
| Mode1 6: Support vector machine |  | 0.661 (0.231, 0.893) | 0.362 (0.118, 0.744) | 1.656 (1.177, 2.503) | 0.502 (0.353, 0.644) | 0.445 (0.315, 0.624) | 0.295 (0.183, 0.49) | 0.372 (0.142, 0.745) | 0.356 (0.189, 0.464) |
| Mode1 7: kNN |  | 0.505 (0.376, 0.655) | 0.628 (0.472, 0.787) | 1.856 (1.542, 2.188) | 0.602 (0.518, 0.657) | 0.594 (0.495, 0.684) | 0.371 (0.272, 0.504) | 0.75 (0.604, 0.83) | 0.399 (0.33, 0.482) |
| Mode1 8: LDA |  | 0.5 (0.357, 0.66) | 0.662 (0.488, 0.805) | 1.839 (1.518, 2.173) | 0.653 (0.627, 0.676) | 0.616 (0.525, 0.698) | 0.405 (0.299, 0.529) | 0.777 (0.715, 0.831) | 0.411 (0.34, 0.471) |
| Mode1 9: QDA |  | 0.491 (0.376, 0.607) | 0.653 (0.507, 0.798) | 1.875 (1.588, 2.116) | 0.648 (0.616, 0.669) | 0.607 (0.519, 0.698) | 0.392 (0.29, 0.498) | 0.767 (0.654, 0.822) | 0.399 (0.323, 0.463) |
| Mode1 1: Logistic regression | Valence and arousal | 0.634 (0.445, 0.755) | 0.624 (0.469, 0.742) | 1.475 (1.146, 1.939) | 0.683 (0.622, 0.721) | 0.628 (0.539, 0.693) | 0.392 (0.277, 0.504) | 0.805 (0.631, 0.869) | 0.468 (0.356, 0.561) |
| Mode1 2: Random Forest |  | 0.651 (0.474, 0.812) | 0.554 (0.394, 0.694) | 1.492 (1.1, 1.953) | 0.655 (0.587, 0.711) | 0.582 (0.483, 0.668) | 0.374 (0.295, 0.478) | 0.811 (0.747, 0.882) | 0.46 (0.352, 0.526) |
| Mode1 3: MLP classifier |  | 0.603 (0.436, 0.752) | 0.527 (0.339, 0.681) | 1.663 (1.265, 2.077) | 0.615 (0.54, 0.691) | 0.549 (0.446, 0.639) | 0.34 (0.257, 0.414) | 0.781 (0.701, 0.864) | 0.419 (0.328, 0.5) |
| Mode1 4: XGB classifier |  | 0.648 (0.5, 0.755) | 0.615 (0.509, 0.725) | 1.44 (1.088, 1.776) | 0.675 (0.618, 0.736) | 0.624 (0.553, 0.684) | 0.412 (0.333, 0.492) | 0.825 (0.777, 0.877) | 0.487 (0.416, 0.569) |
| Mode1 5: Gradient boosting classifier |  | 0.674 (0.438, 0.814) | 0.514 (0.332, 0.693) | 1.464 (1.103, 2.036) | 0.648 (0.574, 0.71) | 0.559 (0.449, 0.666) | 0.368 (0.278, 0.477) | 0.768 (0.489, 0.885) | 0.456 (0.336, 0.541) |
| Mode1 6: Support vector machine |  | 0.647 (0.443, 0.822) | 0.474 (0.248, 0.728) | 1.586 (1.252, 2.054) | 0.601 (0.456, 0.717) | 0.522 (0.395, 0.653) | 0.348 (0.259, 0.465) | 0.611 (0.313, 0.857) | 0.422 (0.318, 0.501) |
| Mode1 7: kNN |  | 0.678 (0.533, 0.815) | 0.606 (0.507, 0.698) | 1.361 (0.998, 1.759) | 0.694 (0.643, 0.748) | 0.626 (0.557, 0.693) | 0.415 (0.342, 0.495) | 0.836 (0.786, 0.888) | 0.5 (0.422, 0.582) |
| Mode1 8: LDA |  | 0.667 (0.569, 0.75) | 0.649 (0.556, 0.725) | 1.351 (1.137, 1.626) | 0.694 (0.661, 0.72) | 0.654 (0.596, 0.693) | 0.439 (0.385, 0.498) | 0.837 (0.797, 0.87) | 0.518 (0.457, 0.566) |
| Mode1 9: QDA |  | 0.694 (0.559, 0.817) | 0.627 (0.524, 0.719) | 1.29 (0.952, 1.667) | 0.706 (0.661, 0.743) | 0.646 (0.582, 0.706) | 0.435 (0.376, 0.511) | 0.846 (0.802, 0.894) | 0.522 (0.453, 0.591) |

**Table H.** Average performance metrics and 95% credible intervals (CIs) for nine machine learning classifiers under repeated 5‑fold nested cross‑validation with 100 independent repetitions with a penalty of $C=3.5$, with outliers removed.

| Model | Predictor | Sensitivity | Specificity | Expected cost | AUC | Accuracy | PPV | NPV | F1 score |
| --- | --- | --- | --- | --- | --- | --- | --- | --- | --- |
| Mode1 1: Logistic regression | Valence | 0.686 (0.41, 0.896) | 0.474 (0.174, 0.748) | 1.626 (1.104, 2.437) | 0.68 (0.629, 0.713) | 0.535 (0.372, 0.667) | 0.325 (0.199, 0.433) | 0.651 (0.321, 0.854) | 0.424 (0.263, 0.505) |
| Mode1 2: Random Forest |  | 0.734 (0.59, 0.874) | 0.459 (0.303, 0.572) | 1.473 (1.096, 1.921) | 0.637 (0.575, 0.692) | 0.536 (0.452, 0.614) | 0.352 (0.304, 0.409) | 0.814 (0.633, 0.884) | 0.466 (0.407, 0.519) |
| Mode1 3: MLP classifier |  | 0.771 (0.564, 0.933) | 0.416 (0.217, 0.569) | 1.385 (0.877, 2.028) | 0.647 (0.566, 0.713) | 0.515 (0.395, 0.596) | 0.345 (0.287, 0.414) | 0.818 (0.526, 0.942) | 0.466 (0.381, 0.542) |
| Mode1 4: XGB classifier |  | 0.728 (0.564, 0.858) | 0.459 (0.341, 0.599) | 1.495 (1.063, 1.977) | 0.643 (0.57, 0.716) | 0.534 (0.466, 0.606) | 0.349 (0.291, 0.401) | 0.821 (0.67, 0.903) | 0.46 (0.394, 0.522) |
| Mode1 5: Gradient boosting classifier |  | 0.784 (0.621, 0.938) | 0.353 (0.158, 0.531) | 1.403 (1.01, 1.881) | 0.612 (0.556, 0.672) | 0.474 (0.359, 0.566) | 0.328 (0.266, 0.382) | 0.763 (0.432, 0.913) | 0.452 (0.366, 0.506) |
| Mode1 6: Support vector machine |  | 0.688 (0.419, 0.886) | 0.379 (0.146, 0.597) | 1.711 (1.233, 2.446) | 0.591 (0.454, 0.684) | 0.467 (0.346, 0.59) | 0.313 (0.211, 0.432) | 0.605 (0.299, 0.823) | 0.404 (0.284, 0.484) |
| Mode1 7: kNN |  | 0.742 (0.602, 0.876) | 0.52 (0.387, 0.649) | 1.383 (0.984, 1.844) | 0.677 (0.618, 0.736) | 0.582 (0.509, 0.667) | 0.389 (0.336, 0.467) | 0.855 (0.77, 0.917) | 0.496 (0.425, 0.564) |
| Mode1 8: LDA |  | 0.673 (0.574, 0.76) | 0.592 (0.484, 0.707) | 1.551 (1.298, 1.839) | 0.691 (0.658, 0.717) | 0.614 (0.539, 0.684) | 0.408 (0.337, 0.489) | 0.832 (0.79, 0.871) | 0.493 (0.428, 0.543) |
| Mode1 9: QDA |  | 0.746 (0.607, 0.831) | 0.559 (0.486, 0.647) | 1.331 (1.043, 1.79) | 0.707 (0.662, 0.739) | 0.611 (0.562, 0.663) | 0.407 (0.34, 0.458) | 0.861 (0.81, 0.908) | 0.515 (0.428, 0.564) |
| Mode1 1: Logistic regression | Arousal | 0.736 (0.45, 0.986) | 0.331 (0.055, 0.658) | 1.593 (0.993, 2.269) | 0.642 (0.601, 0.671) | 0.443 (0.306, 0.624) | 0.308 (0.193, 0.44) | 0.444 (0.15, 0.798) | 0.401 (0.276, 0.487) |
| Mode1 2: Random Forest |  | 0.68 (0.509, 0.858) | 0.351 (0.139, 0.553) | 1.769 (1.322, 2.264) | 0.573 (0.49, 0.631) | 0.443 (0.333, 0.553) | 0.294 (0.223, 0.348) | 0.576 (0.297, 0.803) | 0.395 (0.32, 0.457) |
| Mode1 3: MLP classifier |  | 0.633 (0.438, 0.824) | 0.404 (0.168, 0.614) | 1.882 (1.369, 2.429) | 0.586 (0.502, 0.663) | 0.467 (0.346, 0.592) | 0.299 (0.23, 0.401) | 0.638 (0.375, 0.819) | 0.385 (0.302, 0.465) |
| Mode1 4: XGB classifier |  | 0.663 (0.509, 0.841) | 0.369 (0.19, 0.562) | 1.811 (1.325, 2.26) | 0.567 (0.467, 0.642) | 0.451 (0.36, 0.558) | 0.295 (0.234, 0.364) | 0.613 (0.405, 0.809) | 0.391 (0.315, 0.462) |
| Mode1 5: Gradient boosting classifier |  | 0.726 (0.477, 0.933) | 0.271 (0.059, 0.491) | 1.689 (1.162, 2.331) | 0.544 (0.457, 0.625) | 0.399 (0.293, 0.501) | 0.281 (0.225, 0.349) | 0.441 (0.135, 0.669) | 0.388 (0.301, 0.454) |
| Mode1 6: Support vector machine |  | 0.813 (0.583, 1) | 0.186 (0, 0.482) | 1.467 (0.982, 2.006) | 0.502 (0.353, 0.644) | 0.362 (0.281, 0.511) | 0.278 (0.22, 0.351) | 0.241 (0, 0.512) | 0.394 (0.325, 0.453) |
| Mode1 7: kNN |  | 0.616 (0.44, 0.791) | 0.469 (0.219, 0.694) | 1.874 (1.421, 2.362) | 0.602 (0.518, 0.657) | 0.51 (0.386, 0.628) | 0.333 (0.245, 0.432) | 0.648 (0.398, 0.826) | 0.403 (0.312, 0.48) |
| Mode1 8: LDA |  | 0.642 (0.502, 0.817) | 0.47 (0.279, 0.644) | 1.782 (1.341, 2.194) | 0.653 (0.627, 0.676) | 0.518 (0.419, 0.615) | 0.339 (0.269, 0.427) | 0.759 (0.573, 0.862) | 0.416 (0.338, 0.471) |
| Mode1 9: QDA |  | 0.634 (0.476, 0.8) | 0.471 (0.26, 0.64) | 1.81 (1.397, 2.201) | 0.648 (0.616, 0.669) | 0.516 (0.406, 0.606) | 0.336 (0.249, 0.447) | 0.759 (0.58, 0.859) | 0.411 (0.335, 0.472) |
| Mode1 1: Logistic regression | Valence and arousal | 0.684 (0.485, 0.848) | 0.568 (0.343, 0.717) | 1.538 (1.13, 2.175) | 0.683 (0.622, 0.721) | 0.601 (0.47, 0.68) | 0.384 (0.275, 0.473) | 0.785 (0.501, 0.873) | 0.478 (0.344, 0.554) |
| Mode1 2: Random Forest |  | 0.706 (0.528, 0.838) | 0.476 (0.324, 0.649) | 1.555 (1.178, 2.094) | 0.655 (0.587, 0.711) | 0.54 (0.448, 0.627) | 0.355 (0.3, 0.447) | 0.796 (0.629, 0.884) | 0.458 (0.359, 0.52) |
| Mode1 3: MLP classifier |  | 0.694 (0.507, 0.841) | 0.417 (0.22, 0.594) | 1.653 (1.166, 2.229) | 0.615 (0.54, 0.691) | 0.495 (0.372, 0.615) | 0.325 (0.259, 0.413) | 0.744 (0.492, 0.864) | 0.429 (0.34, 0.505) |
| Mode1 4: XGB classifier |  | 0.689 (0.559, 0.803) | 0.565 (0.431, 0.697) | 1.525 (1.197, 1.879) | 0.675 (0.618, 0.736) | 0.599 (0.518, 0.676) | 0.398 (0.323, 0.474) | 0.829 (0.719, 0.891) | 0.487 (0.415, 0.548) |
| Mode1 5: Gradient boosting classifier |  | 0.74 (0.571, 0.865) | 0.431 (0.269, 0.645) | 1.477 (1.128, 1.987) | 0.648 (0.574, 0.71) | 0.518 (0.4, 0.658) | 0.353 (0.276, 0.445) | 0.73 (0.467, 0.881) | 0.463 (0.377, 0.537) |
| Mode1 6: Support vector machine |  | 0.727 (0.51, 0.92) | 0.374 (0.108, 0.589) | 1.583 (1.135, 2.171) | 0.601 (0.456, 0.717) | 0.473 (0.338, 0.613) | 0.331 (0.261, 0.454) | 0.545 (0.235, 0.84) | 0.43 (0.348, 0.509) |
| Mode1 7: kNN |  | 0.73 (0.597, 0.843) | 0.549 (0.412, 0.685) | 1.397 (1.026, 1.822) | 0.694 (0.643, 0.748) | 0.599 (0.527, 0.689) | 0.4 (0.34, 0.488) | 0.849 (0.791, 0.909) | 0.504 (0.43, 0.575) |
| Mode1 8: LDA |  | 0.688 (0.604, 0.757) | 0.617 (0.506, 0.701) | 1.476 (1.254, 1.734) | 0.694 (0.661, 0.72) | 0.637 (0.561, 0.69) | 0.427 (0.368, 0.48) | 0.84 (0.809, 0.868) | 0.515 (0.461, 0.564) |
| Mode1 9: QDA |  | 0.724 (0.595, 0.843) | 0.597 (0.511, 0.702) | 1.37 (1.004, 1.79) | 0.706 (0.661, 0.743) | 0.633 (0.58, 0.693) | 0.425 (0.37, 0.488) | 0.853 (0.801, 0.907) | 0.524 (0.459, 0.579) |

**Table I.** Average performance metrics and 95% credible intervals (CIs) for nine machine learning classifiers under repeated 5‑fold nested cross‑validation with 100 independent repetitions with a penalty of $C=4$, with outliers removed.

| Model | Predictor | Sensitivity | Specificity | Expected cost | AUC | Accuracy | PPV | NPV | F1 score |
| --- | --- | --- | --- | --- | --- | --- | --- | --- | --- |
| Mode1 1: Logistic regression | Valence | 0.767 (0.532, 0.924) | 0.376 (0.148, 0.606) | 1.555 (1.121, 2.341) | 0.68 (0.629, 0.713) | 0.486 (0.355, 0.598) | 0.327 (0.246, 0.396) | 0.59 (0.3, 0.862) | 0.444 (0.342, 0.516) |
| Mode1 2: Random Forest |  | 0.773 (0.626, 0.881) | 0.397 (0.247, 0.535) | 1.511 (1.12, 2.027) | 0.637 (0.575, 0.692) | 0.503 (0.412, 0.592) | 0.34 (0.29, 0.387) | 0.801 (0.593, 0.899) | 0.463 (0.398, 0.513) |
| Mode1 3: MLP classifier |  | 0.806 (0.602, 0.953) | 0.373 (0.203, 0.519) | 1.403 (0.888, 2.109) | 0.647 (0.566, 0.713) | 0.495 (0.391, 0.578) | 0.338 (0.28, 0.392) | 0.809 (0.499, 0.964) | 0.469 (0.385, 0.534) |
| Mode1 4: XGB classifier |  | 0.758 (0.588, 0.905) | 0.42 (0.244, 0.564) | 1.547 (1.124, 2.12) | 0.643 (0.57, 0.716) | 0.515 (0.425, 0.602) | 0.344 (0.297, 0.392) | 0.806 (0.557, 0.908) | 0.463 (0.39, 0.521) |
| Mode1 5: Gradient boosting classifier |  | 0.825 (0.669, 0.953) | 0.298 (0.133, 0.455) | 1.401 (0.969, 1.926) | 0.612 (0.556, 0.672) | 0.446 (0.351, 0.54) | 0.319 (0.277, 0.368) | 0.731 (0.413, 0.916) | 0.454 (0.398, 0.502) |
| Mode1 6: Support vector machine |  | 0.741 (0.439, 0.907) | 0.321 (0.116, 0.564) | 1.713 (1.191, 2.666) | 0.591 (0.454, 0.684) | 0.439 (0.342, 0.583) | 0.308 (0.231, 0.412) | 0.561 (0.286, 0.837) | 0.414 (0.323, 0.486) |
| Mode1 7: kNN |  | 0.772 (0.609, 0.902) | 0.476 (0.318, 0.615) | 1.434 (0.911, 2.017) | 0.677 (0.618, 0.736) | 0.56 (0.469, 0.658) | 0.379 (0.326, 0.452) | 0.853 (0.673, 0.933) | 0.495 (0.422, 0.566) |
| Mode1 8: LDA |  | 0.703 (0.621, 0.783) | 0.546 (0.449, 0.67) | 1.643 (1.352, 1.932) | 0.691 (0.658, 0.717) | 0.59 (0.526, 0.666) | 0.392 (0.336, 0.47) | 0.832 (0.776, 0.871) | 0.489 (0.428, 0.541) |
| Mode1 9: QDA |  | 0.769 (0.611, 0.86) | 0.529 (0.452, 0.621) | 1.397 (1.092, 1.953) | 0.707 (0.662, 0.739) | 0.596 (0.544, 0.641) | 0.399 (0.339, 0.447) | 0.865 (0.802, 0.909) | 0.514 (0.443, 0.553) |
| Mode1 1: Logistic regression | Arousal | 0.815 (0.528, 1) | 0.237 (0.03, 0.531) | 1.502 (0.993, 2.365) | 0.642 (0.601, 0.671) | 0.398 (0.293, 0.531) | 0.303 (0.229, 0.413) | 0.394 (0.138, 0.753) | 0.417 (0.316, 0.483) |
| Mode1 2: Random Forest |  | 0.774 (0.612, 0.914) | 0.228 (0.05, 0.465) | 1.678 (1.246, 2.256) | 0.573 (0.49, 0.631) | 0.381 (0.289, 0.518) | 0.283 (0.235, 0.342) | 0.436 (0.158, 0.765) | 0.404 (0.331, 0.448) |
| Mode1 3: MLP classifier |  | 0.735 (0.536, 0.896) | 0.281 (0.091, 0.475) | 1.78 (1.246, 2.381) | 0.586 (0.502, 0.663) | 0.408 (0.307, 0.528) | 0.287 (0.231, 0.346) | 0.541 (0.252, 0.803) | 0.4 (0.336, 0.472) |
| Mode1 4: XGB classifier |  | 0.739 (0.567, 0.893) | 0.261 (0.106, 0.439) | 1.783 (1.27, 2.377) | 0.567 (0.467, 0.642) | 0.394 (0.32, 0.509) | 0.281 (0.223, 0.348) | 0.506 (0.258, 0.798) | 0.395 (0.307, 0.455) |
| Mode1 5: Gradient boosting classifier |  | 0.842 (0.664, 0.971) | 0.137 (0.006, 0.316) | 1.493 (1.089, 2.117) | 0.544 (0.457, 0.625) | 0.335 (0.267, 0.44) | 0.274 (0.227, 0.314) | 0.294 (0.038, 0.622) | 0.405 (0.341, 0.448) |
| Mode1 6: Support vector machine |  | 0.869 (0.647, 1) | 0.122 (0, 0.332) | 1.401 (0.963, 2.114) | 0.502 (0.353, 0.644) | 0.332 (0.272, 0.433) | 0.273 (0.222, 0.35) | 0.198 (0, 0.448) | 0.406 (0.33, 0.452) |
| Mode1 7: kNN |  | 0.73 (0.571, 0.879) | 0.312 (0.105, 0.514) | 1.77 (1.291, 2.335) | 0.602 (0.518, 0.657) | 0.429 (0.316, 0.556) | 0.303 (0.226, 0.377) | 0.509 (0.179, 0.785) | 0.408 (0.328, 0.479) |
| Mode1 8: LDA |  | 0.77 (0.588, 0.881) | 0.308 (0.138, 0.499) | 1.613 (1.264, 2.238) | 0.653 (0.627, 0.676) | 0.437 (0.333, 0.544) | 0.309 (0.266, 0.363) | 0.698 (0.377, 0.873) | 0.427 (0.34, 0.479) |
| Mode1 9: QDA |  | 0.759 (0.595, 0.876) | 0.309 (0.151, 0.476) | 1.655 (1.264, 2.186) | 0.648 (0.616, 0.669) | 0.435 (0.338, 0.523) | 0.306 (0.263, 0.358) | 0.698 (0.418, 0.876) | 0.422 (0.36, 0.472) |
| Mode1 1: Logistic regression | Valence and arousal | 0.725 (0.569, 0.87) | 0.517 (0.268, 0.689) | 1.582 (1.154, 2.153) | 0.683 (0.622, 0.721) | 0.575 (0.433, 0.672) | 0.381 (0.282, 0.473) | 0.77 (0.502, 0.883) | 0.484 (0.36, 0.562) |
| Mode1 2: Random Forest |  | 0.753 (0.59, 0.876) | 0.403 (0.246, 0.573) | 1.585 (1.182, 2.13) | 0.655 (0.587, 0.711) | 0.501 (0.395, 0.585) | 0.339 (0.263, 0.414) | 0.764 (0.478, 0.899) | 0.455 (0.373, 0.52) |
| Mode1 3: MLP classifier |  | 0.754 (0.585, 0.893) | 0.329 (0.14, 0.51) | 1.655 (1.226, 2.202) | 0.615 (0.54, 0.691) | 0.448 (0.34, 0.553) | 0.311 (0.258, 0.365) | 0.7 (0.415, 0.862) | 0.429 (0.363, 0.493) |
| Mode1 4: XGB classifier |  | 0.716 (0.583, 0.814) | 0.509 (0.322, 0.653) | 1.627 (1.252, 2.056) | 0.675 (0.618, 0.736) | 0.567 (0.455, 0.65) | 0.378 (0.309, 0.447) | 0.811 (0.631, 0.893) | 0.479 (0.413, 0.549) |
| Mode1 5: Gradient boosting classifier |  | 0.775 (0.611, 0.901) | 0.366 (0.171, 0.58) | 1.534 (1.171, 2.071) | 0.648 (0.574, 0.71) | 0.481 (0.347, 0.61) | 0.337 (0.264, 0.405) | 0.677 (0.335, 0.872) | 0.456 (0.378, 0.521) |
| Mode1 6: Support vector machine |  | 0.78 (0.573, 0.967) | 0.305 (0.066, 0.558) | 1.574 (1.074, 2.216) | 0.601 (0.456, 0.717) | 0.438 (0.298, 0.6) | 0.321 (0.274, 0.432) | 0.495 (0.187, 0.844) | 0.435 (0.372, 0.507) |
| Mode1 7: kNN |  | 0.756 (0.624, 0.871) | 0.51 (0.36, 0.628) | 1.467 (1.056, 1.995) | 0.694 (0.643, 0.748) | 0.578 (0.475, 0.658) | 0.388 (0.329, 0.477) | 0.855 (0.756, 0.915) | 0.501 (0.425, 0.573) |
| Mode1 8: LDA |  | 0.704 (0.624, 0.781) | 0.588 (0.47, 0.679) | 1.594 (1.341, 1.914) | 0.694 (0.661, 0.72) | 0.621 (0.553, 0.676) | 0.416 (0.363, 0.479) | 0.839 (0.79, 0.873) | 0.511 (0.459, 0.563) |
| Mode1 9: QDA |  | 0.74 (0.624, 0.845) | 0.572 (0.492, 0.657) | 1.467 (1.095, 1.854) | 0.706 (0.661, 0.743) | 0.619 (0.548, 0.671) | 0.415 (0.366, 0.478) | 0.856 (0.804, 0.906) | 0.521 (0.455, 0.571) |
